# BHBA attenuates endoplasmic reticulum stress‐dependent neuroinflammation via the gut–brain axis in a mouse model of heat stress

**DOI:** 10.1111/cns.14840

**Published:** 2024-07-07

**Authors:** Yuzhen Sui, Xiao Feng, Yue Ma, Yimeng Zou, Yanli Liu, Jian Huang, Xiaoyan Zhu, Jianguo Wang

**Affiliations:** ^1^ College of Veterinary Medicine Northwest A&F University Yangling Shaanxi China

**Keywords:** endoplasmic reticulum stress, gut‐microbiota, heat stress, Neuroinflammation, β‐Hydroxybutyric acid

## Abstract

**Background:**

Heat stress (HS) commonly occurs as a severe pathological response when the body's sensible temperature exceeds its thermoregulatory capacity, leading to the development of chronic brain inflammation, known as neuroinflammation. Emerging evidence suggests that HS leads to the disruption of the gut microbiota, whereas abnormalities in the gut microbiota have been demonstrated to affect neuroinflammation. However, the mechanisms underlying the effects of HS on neuroinflammation are poorly studied. Meanwhile, effective interventions have been unclear. β‐Hydroxybutyric acid (BHBA) has been found to have neuroprotective and anti‐inflammatory properties in previous studies. This study aims to explore the modulatory effects of BHBA on neuroinflammation induced by HS and elucidate the underlying molecular mechanisms.

**Methods:**

An in vivo and in vitro model of HS was constructed under the precondition of BHBA pretreatment. The modulatory effects of BHBA on HS‐induced neuroinflammation were explored and the underlying molecular mechanisms were elucidated by flow cytometry, WB, qPCR, immunofluorescence staining, DCFH‐DA fluorescent probe assay, and 16S rRNA gene sequencing of colonic contents.

**Results:**

Heat stress was found to cause gut microbiota disruption in HS mouse models, and TM7 and *[Previotella]* spp. may be the best potential biomarkers for assessing the occurrence of HS. Fecal microbiota transplantation associated with BHBA effectively reversed the disruption of gut microbiota in HS mice. Moreover, BHBA may inhibit microglia hyperactivation, suppress neuroinflammation (TNF‐α, IL‐1β, and IL‐6), and reduce the expression of cortical endoplasmic reticulum stress (ERS) markers (GRP78 and CHOP) mainly through its modulatory effects on the gut microbiota (TM7, *Lactobacillus* spp., *Ruminalococcus* spp., and *Prevotella* spp.). In vitro experiments revealed that BHBA (1 mM) raised the expression of the ERS marker GRP78, enhanced cellular activity, and increased the generation of reactive oxygen species (ROS) and anti‐inflammatory cytokines (IL‐10), while also inhibiting HS‐induced apoptosis, ROS production, and excessive release of inflammatory cytokines (TNF‐α and IL‐1β) in mouse BV2 cells.

**Conclusion:**

β‐Hydroxybutyric acid may be an effective agent for preventing neuroinflammation in HS mice, possibly due to its ability to inhibit ERS and subsequent microglia neuroinflammation via the gut–brain axis. These findings lay the groundwork for future research and development of BHBA as a preventive drug for HS and provide fresh insights into techniques for treating neurological illnesses by modifying the gut microbiota.

## Graphical abstract

Heat stress affects the gut microbiota (TM7, *Lactobacillus* spp., *Ruminococcus* spp., and *[Prevotella]* spp.), which in turn acts on the PERK/eIF2α/ATF4/CHOP, TLR4/NF‐κB, and TLR4/p38MAPK signaling pathways to induce endoplasmic reticulum stress (ERS) and ERS‐dependent neuroinflammation, whereas β‐Hydroxybutyric acid exerts a potential therapeutic role in HS‐induced neuroinflammation in mice via the gut–brain axis.

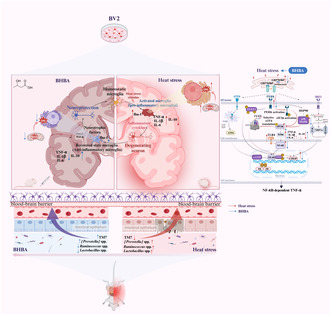

## 1 INTRODUCTION

One characteristic of global climate change in recent years has been the increasing duration, frequency, and intensity of heatwaves, a phenomenon that has led to a significant rise in heat exposure globally, a grim situation that peaked in the summer of 2022 with a total of 61,000 heat‐related fatalities in 35 European countries.^1^, ^2^ Heat stress (HS) arises when the body's sensible temperature surpasses its thermoregulatory capacity, adversely affecting health and growth.^3^ Numerous preceding studies have emphasized the high sensitivity of the central nervous system (CNS) to HS.^4^ Exposure to HS can have significant effects on brain structure and function, including neuronal loss, convulsions, neurogenic deficits, memory impairment, cognitive impairment, and neuroinflammation.^5^, ^6^ It is widely recognized that bidirectional communication between the brain and the gut occurs through the gut microbiota.^7^ Emerging evidence suggests that the gut–brain axis plays a crucial role in regulating central nervous system function and may be involved in treating multiple sclerosis, Parkinson's disease, Alzheimer's disease, and postoperative cognitive dysfunction.^8^, ^9^, ^10^, ^11^ Specifically, dysbiosis affects not only gut‐intrinsic processess but also the production of bacterial metabolites and hormones that modulate the function of distal tissues such as the CNS.^11^, ^12^ The effects of HS on the gut microbiota are primarily manifested in altered bacterial composition and metabolite levels, and the gut microbiota is affected by the direct stimulation of the vagus nerve through the indirect modulation of microglial cells, a key participant in neuroinflammation, and the direct stimulation of the vagus nerve.^3^, ^13^, ^14^ The exact mechanisms by which HS induces neuroinflammation remain to be elucidated, and the therapeutic effects of neuroinflammation are far from satisfactory; therefore, there is an increasing focus on utilizing the potential of the gut microbiome as a promising target for therapeutic neuroinflammatory interventions.^15^ Transcriptome sequencing analysis has demonstrated that HS induces the endoplasmic reticulum stress (ERS) response/unfolded protein response (UPR), specifically activating the pro‐apoptotic pathway involving transcription factors (ATF4) and CEBP homologous protein (CHOP).^16^ Furthermore, HS has been found to initiate ERS and trigger the phosphorylation of eukaryotic initiation factor 2α (p‐eIF2α), leading to CHOP activation.^17^ This signaling cascade compromises the integrity of the epithelial barrier by inducing apoptosis in intestinal epithelial cells. Meanwhile, ERS is also an important factor contributing to the deterioration of neurological disorders. Research on neurodegenerative diseases has revealed that the buildup of misfolded proteins in neurons, coupled with ERS, can impair neuronal function.^18^ Mounting evidence indicates that ERS‐triggered activation of inflammatory vesicles contributes to the pathogenesis of various inflammatory conditions, while the ERS response in neighboring glial cells can generate aberrant inflammatory signals, exacerbating disease progression.^19^, ^20^ Although the precise processes underlying ERS‐mediated neuroinflammation remain unclear, inhibiting ERS‐dependent neuroinflammation may be a potential therapeutic strategy to prevent HS.

Microglia play a pivotal role in the brain's immune function, influencing neuronal wiring, synaptic plasticity, phagocytosis, and the support of neurons and neuronal progenitors through growth factor secretion, while their uncontrolled overactivation is a major component of neuroinflammation, with ample evidence implicating microglia in neurodegeneration across various chronic neuroinflammatory and neurodegenerative disorders.^21^, ^22^, ^23^, ^24^, ^25^ Previous studies on the neurological effects of HS have focused on the subcortical hippocampus, while relatively few studies have been conducted on the cerebral cortex.^1^, ^6^, ^26^ In fact, the cerebral cortex stands as the most evolutionarily intricate structure in the mammalian brain, encompassing diverse physiological functions, including attention, cognition, learning, and memory.^27^ The cerebral cortex also plays an important role in thermoregulation, in which the body can regulate body temperature through conditioned reflexes to adapt the body to the survival environment.^28^, ^29^ Therefore, it is valuable to supplement the data related to the cerebral cortex under HS.

β‐Hydroxybutyric acid (BHBA) is an important intermediate in the catabolism of amino acids and fatty acids. As a water‐soluble endogenous small‐molecule ketone body, it readily crosses the blood–brain barrier. It has been demonstrated to be neuroprotective in diseases such as Alzheimer's, Parkinson's, anxiety, hypoglycemia, and HS.^22^, ^30^, ^31^, ^32^ A recent study has shown that BHBA can improve hypoglycemia‐induced neuroinflammation by regulating the gut microbiota.^33^ The complex interactions between BHBA and the gut microbiota, and how this vast network affects human and animal health, have been hot research topics. However, whether BHBA can reduce the damage caused by heat stress through gut microbiota modulation has not been well confirmed.

In this study, we investigated whether BHBA has the potential to inhibit HS and the specific mechanism by which BHBA exerts a protective effect on neuroinflammation. Our study indicates that BHBA may be a potential candidate compound for the prevention of HS‐associated neurological damage and bridges a gap in the understanding that BHBA reduces HS‐associated neuroinflammation by modulating the gut microbiota.

## 2 MATERIALS AND METHODS

### 2.1 Chemicals and reagents

β‐Hydroxybutyric acid (BHBA) (#54,965; purity ≥ 99.0%), propidium iodide (PI), and DAPI were obtained from Sigma‐Aldrich (St. Louis, MO, USA). Evo M‐MLV Reverse transcription kits and fluorescence quantification kits were provided by Accurate Biotechnology. Reactive oxygen species assay kit, dimethyl sulfoxide, 3‐[4,5‐dimethylthiazol‐2‐yl]‐2,5‐diphenyltetrazolium bromide (MTT), protease and phosphatase inhibitors were sourced from Solarbio Science & Technology Co Ltd. (Beijing, China). Antibodies utilized for Western blots and immunostaining, including rabbit anti‐nuclear transcription factor‐kappa B (NF‐κB), mouse anti‐heat shock protein (HSP70), rabbit anti‐toll‐like receptor 4 (TLR4), rabbit anti‐eukaryotic initiation factor 2a (eIF2a), rabbit anti‐p‐eIF2a, rabbit anti‐ionized calcium‐binding adapter molecule 1 (Iba1), and FITC‐goat anti‐mouse IgG (H + L) 488, were acquired from Abcam (Cell Signaling, Boston, MA, USA). Additionally, antibodies such as rabbit anti‐P38, rabbit anti‐phosphorylated P38, rabbit anti‐PKR‐like ER Kinase (PERK), goat anti‐mouse IgG antibody, and horseradish peroxidase‐conjugated goat anti‐rabbit IgG antibody were obtained from Cell Signaling Technology (Danvers, MA, USA), while mouse anti‐β‐actin, rabbit anti‐CHOP, rabbit anti‐glucose‐regulated protein 78 (GRP78), and rabbit anti‐ATF4 were sourced from BOSTER (China).

### 2.2 Animals and animal ethics

Male imprinting control region mice aged 2 months were bought from Xi'an Jiaotong University's Laboratory Animals Center (Xi'an, China). Mice were housed in a 12‐hour light and dark cycle, with a temperature maintained between 22°C and 24°C and a humidity level of 60% ± 10%. All activities complied with Northwest A&F University's Guide for Care and Experimental of Laboratory Animals and the Animal Care Commission of the College of Veterinary (certificate no.: SCXK [SHAAN] 2017‐003).

### 2.3 Experimental design and sample collection

**FIGURE 1:**
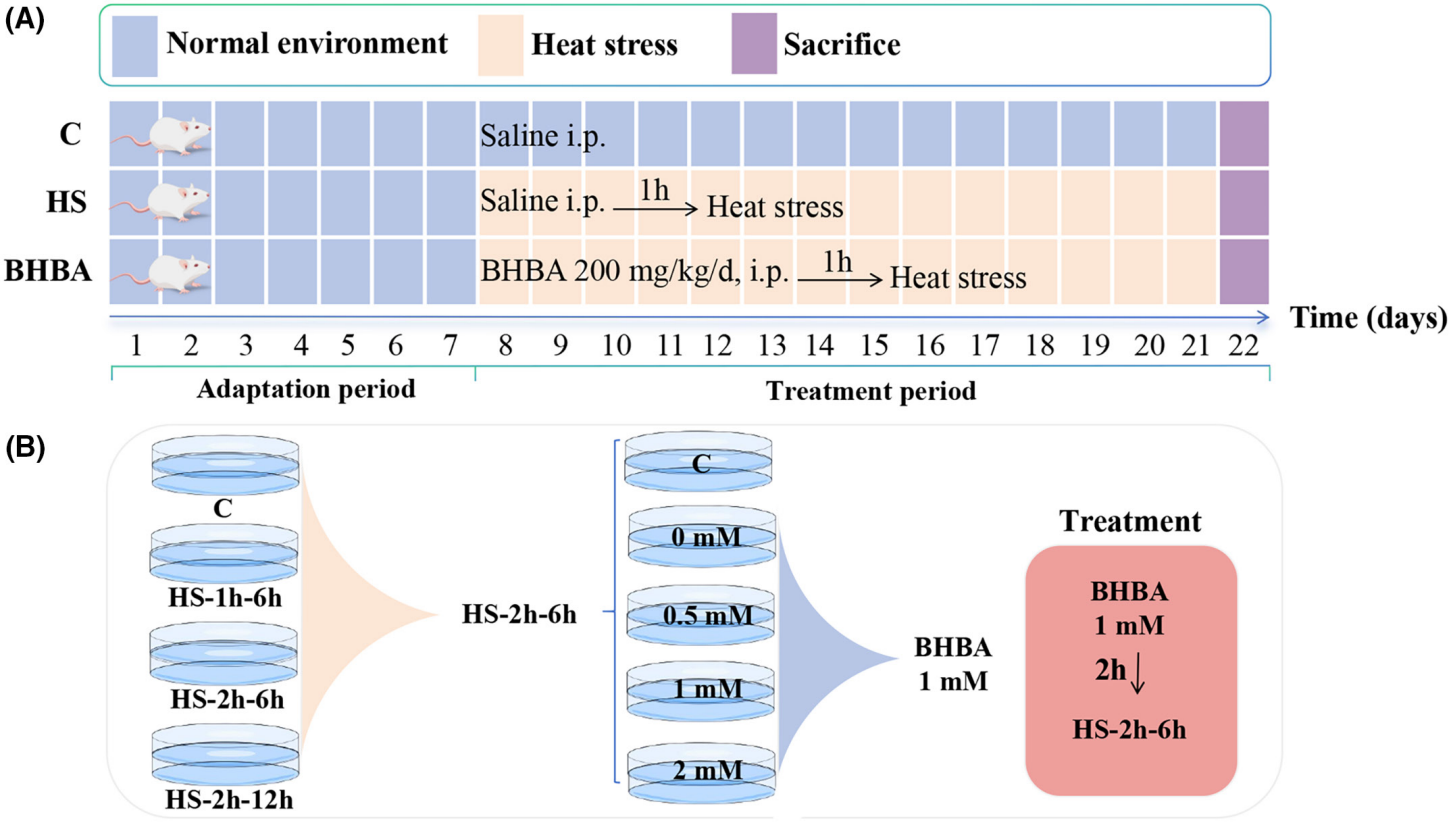
Experimental design diagram. (A) Experiments in vivo. (B) Experiments in vitro.

A 20 mg/mL concentration of BHBA was dissolved in sterile saline. The mice were randomly assigned to three groups (*n* = 30): saline (10 mL/kg/day) for the C group, HS + saline (10 mL/kg/day) for the HS group, and 200 mg/kg/day of BHBA for the BHBA group. The prior study was used to determine the BHBA dosage.^31^, ^34^ Following a week of adaptation, mice were moved to a chamber with constant temperature (43°C) and humidity (60% ± 10%) for 15 min every day for 14 days, starting an hour after the drug was administered (Figure 1A). To minimize the impact of diurnal variation, all procedures commenced at 9:00 a.m. daily. Mice were euthanized the day following the final HS treatment, and samples of the cerebral cortex and colon contents were collected and stored at −80°C for subsequent experiments.

### 2.4 Fecal microbiota transplantation

Fecal samples (BHBA 200 mg/kg/day without HS treatment) were collected starting on the 10th day of BHBA intraperitoneal injection for 4 consecutive days, and the collected fecal microbiota was transplanted to the fecal microbiota transplantation (FMT) group (*n* = 6) by gavage. Fecal transplantation continued for 14 days, and HS was applied to the FMT group 1 h after fecal transplantation. Fresh feces were collected, placed in sterile, precooled phosphate‐buffered saline containing 20% glycerol, and kept at −80°C until further processing.

### 2.5 Cell culture

BV2 microglial cells, derived from Raf/Myc‐immortalized murine neonatal microglia, were procured from the American Type Culture Collection (ATCC, USA) and cultured in Dulbecco's Modified Eagle Medium (DMEM) supplemented with 10% fetal bovine serum (12664025, Gibco, USA) and 1% antibiotics. Cultures were maintained in a 5% CO_2_ incubator at 37 °C. Upon reaching >80% confluence, cells were dissociated using 0.25% trypsin. Cells in the logarithmic growth phase were utilized for subsequent experiments. The design of the partial cell experiments is shown in Figure 1B, with an HS temperature of 43°C.

### 2.6 Cell viability assay

Cell viability was assessed using the Cell Proliferation Kit I according to the manufacturer's protocol (Cat #11465007001, Sigma‐Aldrich, Castle Hill, NSW, Australia). Cells were seeded at a density of 1 × 104 cells/well in 96‐well plates. Each well‐received DMEM was supplemented with 0.5 mg/mL MTT (Sigma‐Aldrich). After 4 h of incubation at 37°C, the medium was aspirated, and 100 μL of dimethyl sulfoxide was added to each well. The formazan product generated from the cleavage of the yellow tetrazolium salt MTT was quantified using a spectrophotometer, measuring absorbance change at 550–600 nm with a microplate reader.

### 2.7 PI staining

BV‐2 microglial cells were plated in a 12‐well plate at a density of 2.6 × 103 cells/cm^2^. Following treatments, cells were exposed to freshly prepared PI solution (10 μg/mL, Sigma) for 15 min at 37°C. Subsequently, cells were fixed with 4% paraformaldehyde supplemented with 4% sucrose and stained with Hoechst 33342 (Invitrogen, #H1399, 1:2000) for 15 min at 37°C. Imaging was performed using an inverted Axiovert 200 M fluorescence microscope controlled by Axiovision software. Cell counting was conducted using Fiji software, and the percentage of PI‐positive cells was determined.

### 2.8 Measurement of intracellular ROS

BV2 cells were seeded in 24‐well plates with a density of 5 × 104 cells/well and cultured in LG‐CM until a better morphology of the cells. The HS group had a 2‐hour treatment at 43°C. Before treatment at 43°C for 2 h, the BHBA group was treated with the most suitable concentration of BHBA (1 mM) for 2 h. The production of intracellular reactive oxygen species (ROS) was measured using fluorescent probes (DCFH‐DA) in accordance with the instruction manual method. Cells were treated for 20 min in DMEM with 10 μM DCFH‐DA. Then, the images were taken by choosing the random field of view (*n* = 20 per group) under fluorescence microscopy and analyzed using Image J.

### 2.9 Flow cytometry

The Dead Cell Apoptosis Kit with Annexin V Alexa Fluor 488 and PI (#V13241; ThermoFisher Scientific) was used to identify apoptosis and necrosis utilizing differential staining with annexin V (early and late apoptotic cells) and PI (necrotic cells exclusively), as directed by the manufacturer. Cells were stained for 15 min with Alexa Fluor 488 Annexin‐V and PI before being examined by flow cytometry. Compensation for fluorescence photobleaching and gating was achieved using unstained and single‐stained controls. Flow cytometry data was analyzed using the FlowJo program.

### 2.10 Immunofluorescence staining

Mice were anesthetized with 0.56% sodium pentobarbital and then exposed to continuous cardiac perfusion with 0.9% saline followed by 4% paraformaldehyde (pH 7.4) until clear liquid flowed out from the right atrial appendage. Acquired brain tissues were preserved in 4% paraformaldehyde, while 50 μm‐thick coronal brain sections were later obtained using a vibrating slicer (VT 1000S, Leica, Wetzlar, Germany). Sections containing cerebral cortex were picked and incubated with primary antibody (rabbit anti‐Iba1, ab178847, 1:500, Abcam) at 4°C overnight. Subsequently, the sections were subjected to secondary antibody (Alexa Fluor 488 goat anti‐rabbit IgG, ab150077, 1:500, Abcam) incubation for 2 h at room temperature protected from light. The cell nuclei were stained with DAPI for 20 min at room temperature. The results were observed under a confocal microscope (Leica, TCS, SP8) after mounting the sections in fluorescent fixative (Dako).

### 2.11 Quantitative RT‐PCR analysis

Total RNA was extracted from the mouse cerebral cortex using TRIzol reagent (Invitrogen, Lafayette, CO, USA). The Evo M‐MLV Reverse Transcription Kit was used to complete cDNA synthesis with 2.0 μg of total RNA. The generated cDNA served as a template for fluorescence quantitative PCR (RT‐PCR), which was carried out on the Bio‐Rad CFX 96TM Real‐Time PCR Detection System (Bio‐Rad, California, USA) using NovoStart® SYBR qPCR SuperMix Plus (Novoprotein Scientific Inc., Nanjing, China). The primer sequences are displayed in Table S1 in Appendix S1, GAPDH was used as an internal control for normalization, and the 2‐ΔΔCt method was used to determine the relative mRNA expression.

### 2.12 Western blot analysis

Protein concentrations in the cerebral cortex were measured using the BCA Protein Concentration Assay Kit according to the manufacturer's instructions. Extracted proteins were separated by SDS‐polyacrylamide gel electrophoresis and then transferred to a PVDF membrane. The membranes were closed at room temperature and incubated overnight at 4°C with primary antibodies: anti‐β‐actin, anti‐HSP70, anti‐TLR4, anti‐p‐38, anti‐p‐p38, anti‐NF‐κB, anti‐PERK, anti‐GRP78, anti‐CHOP, anti‐eIF2a, anti‐p‐eIF2a, and anti‐ATF4. The membranes were then washed five times with TBST and incubated with secondary antibodies (goat anti‐mouse or goat anti‐rabbit) for 2 h. The membranes were detected with enhanced chemiluminescence detection reagents and imaged with a chemiluminescence imager, and then the optical density of the bands was analyzed using Q9 Alliance software.

### 2.13 16S rRNA gene sequence analysis

Microbial genomic DNA was extracted from 6 fecal samples in each group using a QIAamp DNA tool mini kit (Qiagen, Hilden, Germany) following the manufacturer's instructions. Nuohe Zhiyuan Technology Co., Ltd. (Tianjin, China) amplified the V4 and V5 sections of 16S rRNA genes using bacterial primers 515 (forwards: 5′‐GTGCCAGCMGCCGCGGTAA‐3′) and 907 (reverse: 5′‐CCGTCAATTCCTTTGAGTTT‐3′). With the use of the QIAquick PCR Purification Kit (Qiagen) and the Invitrogen dsDNA Detection Kit (Carlsbad, CA, USA), amplified DNA extracted from 2% agarose gels was purified and quantified. Following the building of the DNA libraries, sequencing was carried out using the Illumina MiSeq 250 platform. Raw Illumina read data was uploaded to the NCBI Sequence Read Archive (PRJNA 1071825), and associated data was analyzed using the Gene Cloud tool (https://www.genescloud.cn) and the online website (https://www.omicstudio.cn).

### 2.14 Statistical analysis

Shapiro–Wilk test to test whether the data satisfy the normal distribution (Gaussian distribution). For data not normally distributed, the nonparametric test methods of Mann–Whitney U test and Kruskal–Wallis test were performed according to the grouping, respectively; data satisfying the normal distribution were subjected to subsequent variance chi‐square tests for further significance analysis (*p* > 0.05). Two‐tailed Student's *t‐*tests and one‐way ANOVA with LSD (Least Significant Difference) post hoc tests were used to analyze cerebral cortex and BV2‐related indicators by SPSS (version 21.0, IBM, USA) and GraphPad Prism (version 8.0.2, San Diego, CA, USA). Gut microbiota diversity was analyzed using the Mann–Whitney U‐test and Kruskal–Wallis one‐way ANOVA with LSD post hoc test. Spearman correlation analysis was used to generate heat maps on the https://www.omicshare.com/tools/Home/Soft/getsoft website. All data were presented as mean ± SEM. *p* < 0.05 was considered statistically significant.

## 3 RESULTS

### 3.1 BHBA alleviates HS and inhibits the overactivation of microglia in HS animals and cell models

**FIGURE 2:**
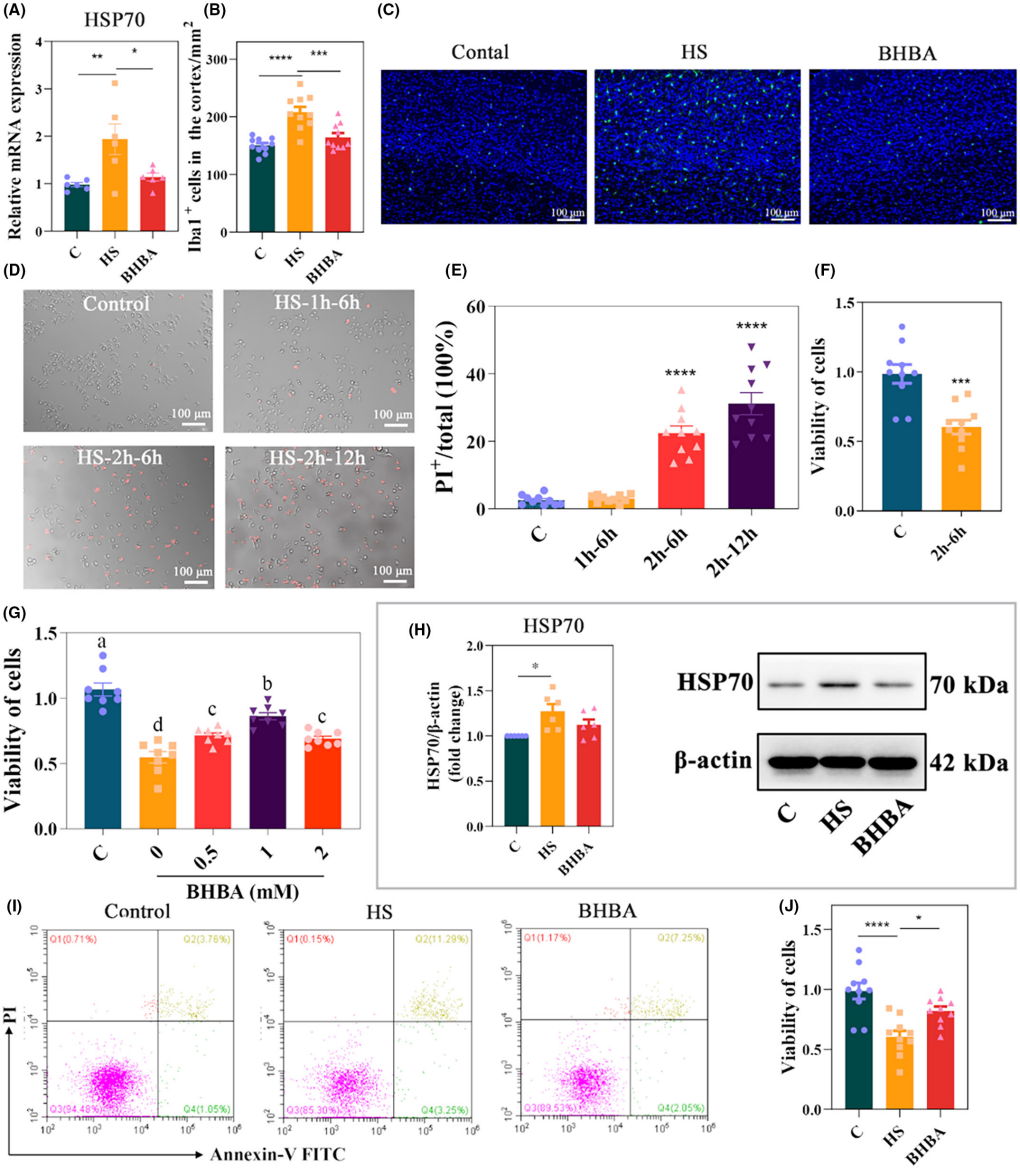
BHBA inhibits overactivation of microglia in HS animals and cell models. (A) The mRNA level of HSP70 in the cerebral cortex (*n* = 6). (B, C) The density of Iba1^+^ cells in the cerebral cortex (40×). (D) PI‐positive BV2 cells. Cells were stained with PI (red) (40×). (E) Statistical analysis of the ratio of PI‐positive cells. (F) Cell viability, measured by MTT assay. Cells were cultured for 6 h after HS for 2 h. (G) Cell viability, measured by MTT assay. Cells were treated with varying BHBA concentrations (0, 0.5, 1, and 2 mM) for 2 h before HS for 2 h, and MTT was assayed after 6 h of incubation. (H) Relative protein expression of HSP70 and representative Western blots of HSP70 in BV2 cells with β‐actin as the loading control (*n* = 6). (I) Flow cytometry analysis of BV2 cells. (J) Cell viability, measured by MTT assay. Cells in the BHBA group were treated with BHBA (1.0 mM) for 2 h before HS for 2 h, and MTT was assayed after 6 h of incubation. *p < 0.05, **p < 0.01, ***p < 0.001, and ****p < 0.0001. All data are expressed as the mean ± SEM. BHBA, β‐Hydroxybutyric acid; HS, heat stress; MTT, 3‐[4,5‐dimethylthiazol‐2‐yl]‐2,5‐diphenyltetrazolium bromide; PI, propidium iodide.

HSP70, the most widely studied and conserved family of heat shock proteins, is rapidly synthesized in response to stress and exerts a protective tolerance to stress, and some past reports have shown that its level is significantly elevated in heat‐stressed mice.^31^, ^35^ We monitored the HSP70 level on day 15. In Figure 2A, HSP70 levels were significantly higher in the HS group than in the C group (*p <* 0.01), while BHBA resulted in a significant decrease in HSP70 mRNA expression (*p <* 0.05). The microglia marker Iba1 in the cerebral cortex of mice was detected by immunofluorescence. Notably, HS mice had a significantly higher density of Iba1+ cells in the cerebral cortex compared with group C, while the BHBA group had a significantly lower density, suggesting that BHBA has an inhibitory effect on the microglia activation in the cerebral cortex under the effect of HS (Figure 2B,C, *p <* 0.05).

Next, we evaluated the effect of HS on BV2 cell apoptosis and cell viability. The PI and MTT results showed that HS for 2 h, and culture for 6 h significantly affected the cellular state of BV2 cells (*p <* 0.0001 and *p <* 0.001, respectively; Figure 2D–F). To investigate whether BHBA could protect against HS‐induced BV2 cell damage, BV2 cells were pretreated with BHBA (0, 0.5, 1.0, and 2.0 mM) for 2 h, heat‐stressed for 2 h, and cultured for 6 h. After 6 h of incubation, cell viability was detected by MTT, and cell viability of BV2 cells pretreated with 1.0 mM BHBA prior to the stimulation of HS increased significantly compared to the HS group and closer to the C group (Figure 2G). Therefore, we used BHBA (1.0 mM) pretreatment for 2 h, HS for 2 h, and incubation for 6 h for subsequent experiments. We discovered that HS stimulation increased HSP 70 (*p <* 0.05) and the rate of early‐stage apoptosis, and significantly decreased cell viability (*p <* 0.0001) compared to group C, whereas BHBA‐pretreated BV2 cells showed reduced HSP70 expression (*p >* 0.05), reduced the rate of early‐stage apoptosis, and increased cell viability (*p <* 0.05) compared with the HS group (Figure 2H–J; Figure S2h in Appendix S2). Based on these results, BHBA may be a potential drug to alleviate HS and inhibit HS‐induced microglia overactivation.

### 3.2 BHBA inhibits inflammatory signaling in HS animals and cell models

**FIGURE 3:**
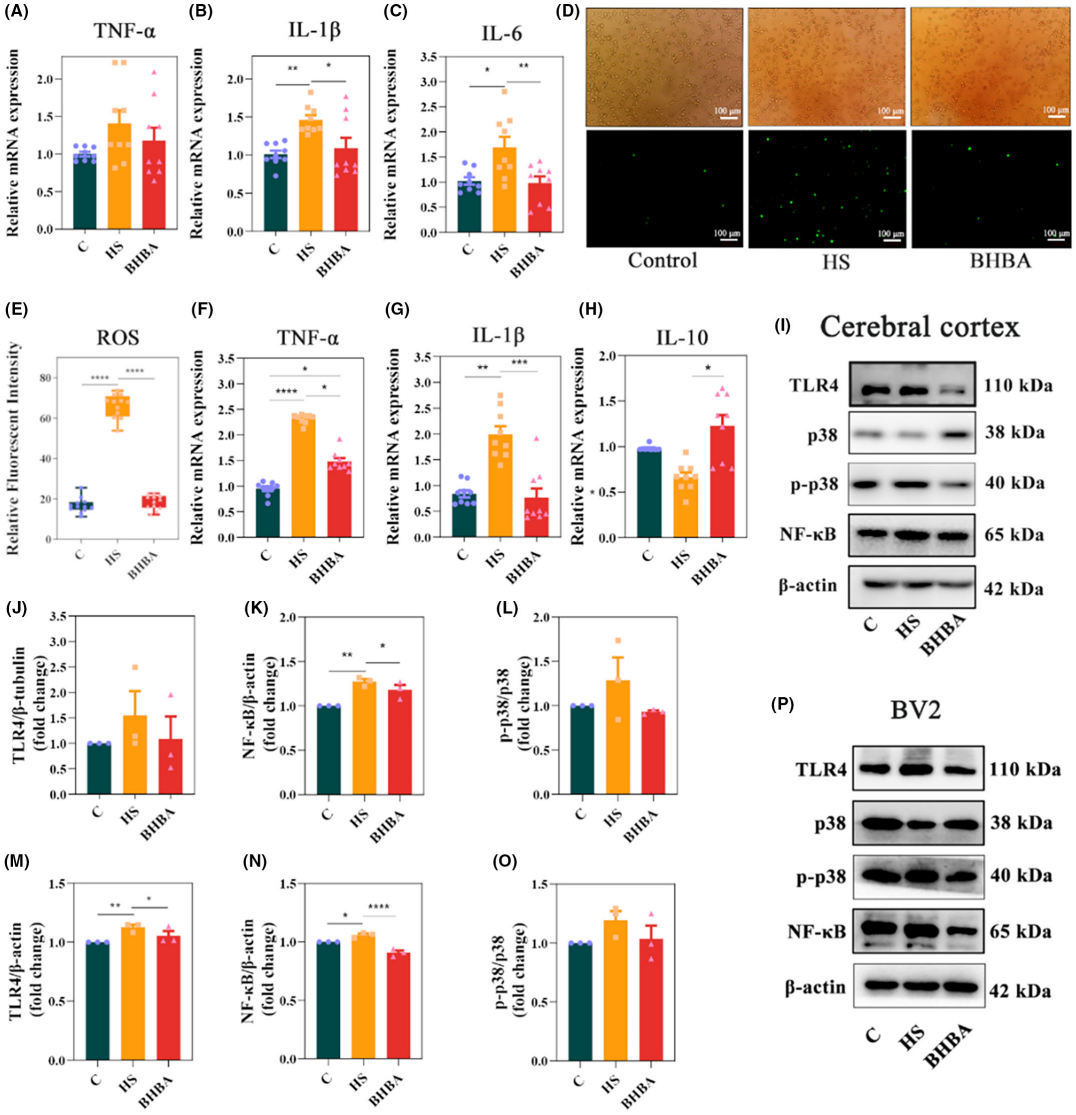
BHBA inhibits inflammatory signaling in HS animals and cell models. (A–C) The mRNA levels of TNF‐α, IL‐1β, and IL‐6 in the cerebral cortex (*n* = 9). (D, E) The level of ROS in BV2 cells (40×). (F–H) The mRNA levels of TNF‐α, IL‐1β, and IL‐10 in BV2 cells (*n* = 9). (I) Representative Western blots of TLR4, p38, p‐p38, and NF‐κB in cerebral cortex with β‐actin as the loading control. (J–L) Relative protein expression of TLR4, NF‐κB, and p‐p38/p38 in cerebral cortex (*n* = 3). (M–O) Relative protein expresssion of TLR4, NF‐κB, and p‐p38/p38 in BV2 cells (*n* = 3). (P) Representative Western blots of TLR4, p38, p‐p38, and NF‐κB in BV2 cells with β‐actin as the loading control. *p < 0.05, **p < 0.01, ***p < 0.001, and ****p < 0.0001. All data are expressed as the mean ± SEM. BHBA, β‐hydroxybutyric acid; HS, heat stress; ROS, reactive oxygen species.

Studies have indicated that microglia activation triggers the production of multiple inflammatory mediators, culminating in hemorrhage and necrosis across several organs, including the heart, liver, and brain.^36^, ^37^ In this study, we assessed the expression levels of inflammatory cytokines in HS mice. According to the results, tumor necrosis factor‐α (TNF‐α) (*p >* 0.05), interleukin‐1β (IL‐1β) (*p <* 0.01), and interleukin‐6 (IL‐6) (*p <* 0.05) increased in HS mice, which could be restored in BHBA‐treated mice (*p >* 0.05, *p* < 0.05, and *p* < 0.01, respectively) (Figure 3A–C). Elevated levels of ROS have been linked to the pathogenesis of various inflammatory diseases, affecting physiological and pathophysiological conditions via inflammatory signaling pathways.^38^, ^39^ Therefore, we detected the level of ROS and found that HS significantly increased its expression, which can be reversed by BHBA treatment (Figure 3D,E; *p <* 0.0001 for both). Consistent alterations in TNF‐α and IL‐1β levels were similarly noted in heat‐stressed BV2 cells (Figure 3F,G). While the expression of anti‐inflammatory cytokine interleukin‐10 (IL‐10) (*p >* 0.05) was reduced in heat‐stressed BV2 cells, BHBA treatment significantly increased the expression of IL‐10 (*p <* 0.05) (Figure 3H). These results indicate that BHBA exerts an inhibitory influence on neuroinflammation in both heat‐stressed animal models and cell models.

To elucidate the inhibitory mechanism of BHBA on neuroinflammation, we investigated classical inflammatory pathways both in vivo and in vitro. In the cerebral cortex tissues of HS mice, the expression of TLR4 (*p >* 0.05), NF‐κB (*p <* 0.01), and *p*‐p38/p38 (*p >* 0.05) was upregulated, whereas in BHBA‐treated mice, these protein levels were decreased (*p >* 0.05, *p <* 0.05, and *p >* 0.05, respectively) (Figure 3I–L; Figure S3i in Appendix S2). Similar results of TLR4, NF‐κB, and *p*‐p38/p38 were also observed in HS‐treated BV2 cells (Figure 3M–P; Figure S3p in Appendix S2). These findings suggest that BHBA exerts inhibitory effects on neuroinflammation in both HS mice and HS‐treated BV2 cells by TLR4/NF‐κB and TLR4/p38 MAPK signaling pathway.

### 3.3 BHBA inhibits ERS signaling in HS animals and cell models

**FIGURE 4:**
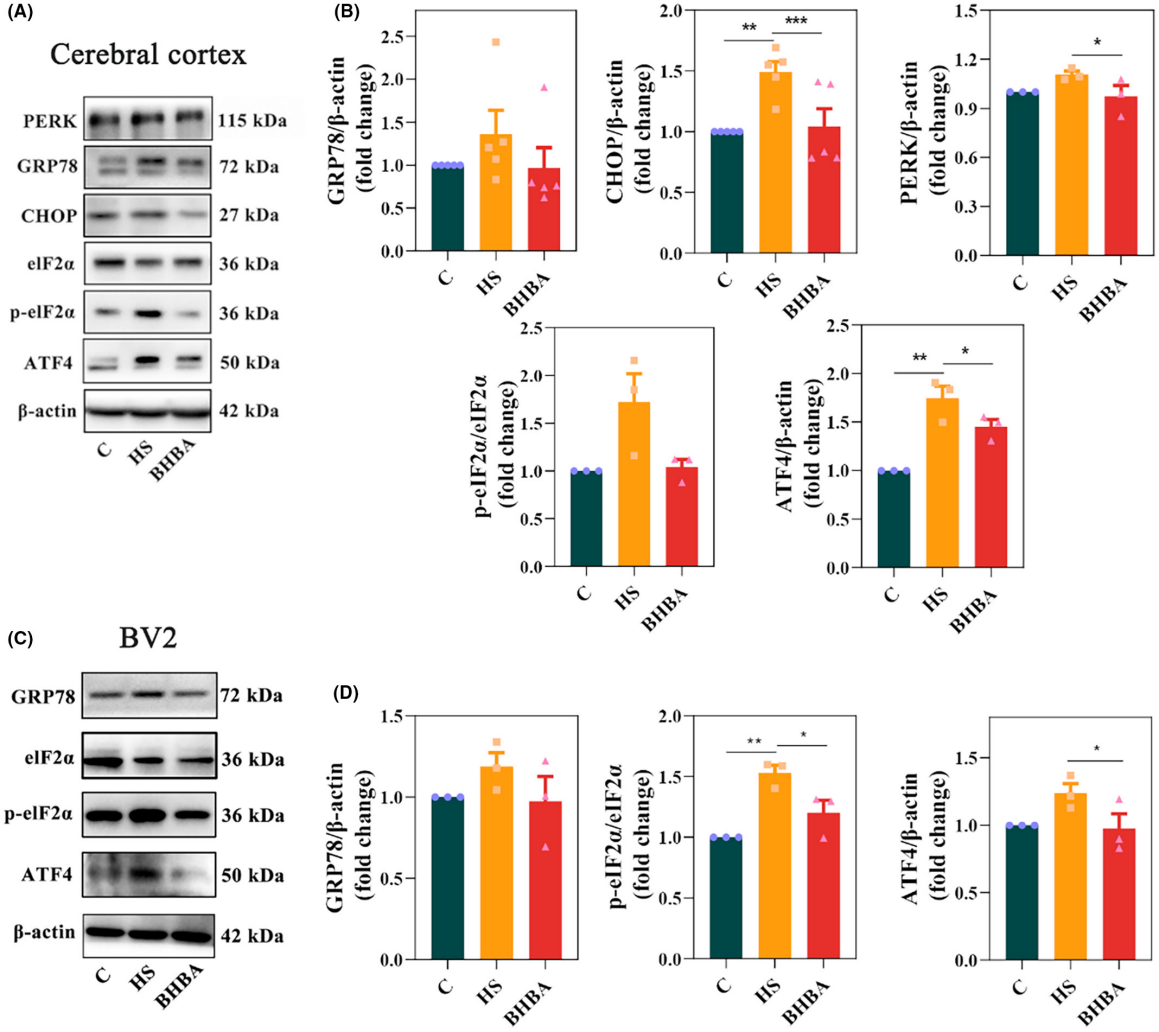
BHBA inhibits ERS signaling in HS animals and cell models. (A) Representative Western blots in cerebral cortex (PERK, GRP78, CHOP, eIF2α, p‐eIF2α, and ATF4) with β‐actin as the loading control. (B) Relative protein expression of GRP78 (*n* = 5), CHOP (*n* = 5), PERK (*n* = 3), p‐eIF2α/eIF2α (*n* = 3), and ATF4 (*n* = 3) in the cerebral cortex. (C) Representative Western blots in BV2 cells (GRP78, eIF2α, p‐eIF2α, and ATF4) with β‐actin as the loading control. (D) Relative protein expression of GRP78 (*n* = 3), p‐eIF2α/eIF2α (*n* = 3), and ATF4 (*n* = 3) in the BV2 cells. *p < 0.05, **p < 0.01, and ***p < 0.001. All data are expressed as the mean ± SEM. BHBA, β‐Hydroxybutyric acid; ERS, endoplasmic reticulum stress; HS, heat stress.

HS can activate the ERS response/UPR.^16^ We detected the classic ERS‐related proteins in the cerebral cortex and BV2 cells to investigate whether BHBA alleviates HS‐induced ERS. Figure 4A,B indicate that HS animals had higher levels of GRP78 (*p >* 0.05) and CHOP (*p <* 0.01) in the cerebral cortex, whereas BHBA‐treated mice had lower levels of GRP78 (*p >* 0.05) and CHOP (*p <* 0.001) compared to HS mice (Figure S4a in Appendix S2). To demonstrate the link between excessive microglial activation and ERS, we detected the ERS‐related marker GRP78 in BV2 cells. The results showed that HS also increased the expression of GRP78 in BV2 cells, while BHBA decreased its expression (Figure 4C,D; Figure S4c in Appendix S2; *p >* 0.05 and *p <* 0.05, respectively). These results suggest that BHBA attenuates ERS signaling molecules in both the HS animal model and cell model.

To investigate the mechanism of ERS inhibition by BHBA, we detected cortical PERK levels along with in vivo and in vitro levels of *p*‐eIF2α/eIF2α and ATF4.^40^ The results revealed elevated expression levels of PERK (*p >* 0.05), the *p*‐eIF2α/eIF2α ratio (*p >* 0.05), and ATF4 (*p <* 0.01) in the cerebral cortex of heat‐stressed (HS) mice, whereas BHBA‐treated mice exhibited reduced expression of PERK (*p >* 0.05), the *p*‐eIF2α/eIF2α ratio (*p* > 0.05), and ATF4 (*p* < 0.05) compared to the HS group (Figure 4A,B). Similar results were also observed in BV2 cells (Figure 4C,D). In conclusion, the above results indicated that BHBA could inhibit HS‐induced ERS by suppressing the PERK/eIF2α/ATF4/CHOP signaling pathway.

### 3.4 BHBA reverses HS‐induced changes in the gut microbiota

**FIGURE 5:**
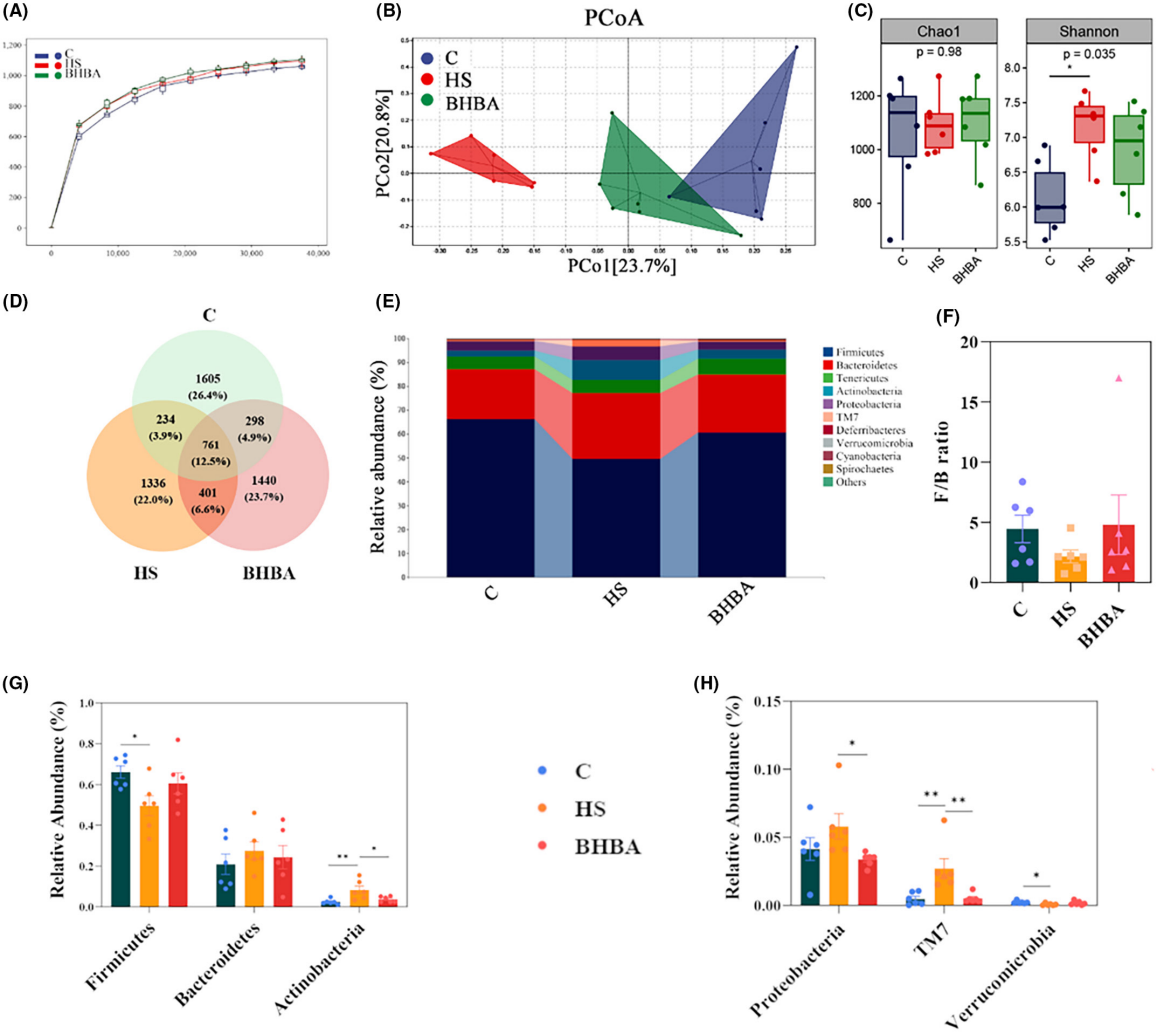
BHBA protects HS‐induced abnormal changes in the gut microbiota at the phylum level. (A) Rarefaction curve. (B) PCoA based on the Bray‐Curtis distance matrix of ASVs. (C) Alpha diversity analysis (Chao1 and Shannon) of gut microbiota. (D) Venn diagram of ASVs. (E) Relative abundance of gut microbial composition at the phylum level (top 10). (F–H) The effects of HS and BHBA on the relative abundance of gut microbiota at the phylum level (F/B ratio, Firmicutes, Bacteroidetes, Actinobacteria, Proteobacteria, TM7, and Verrucomicrobia, respectively). *p < 0.05 and **p < 0.01. All data are expressed as the mean ± SEM, *n* = 6. BHBA, β‐Hydroxybutyric acid; HS, heat stress.

It has been shown that in heat‐stressed animals, dermal vasodilatation leads to reduced intestinal blood supply and insufficient oxygen supply and triggers inflammatory bowel disease and intestinal damage, which in turn can adversely affect colonic gut microbiota homeostasis.^41^, ^42^ Hence, our study assessed the impact of HS on the gut microbiota by analyzing colon contents. The species sparsity curve has leveled off, indicating that the sequencing depth has essentially covered all species in the sample and is sufficient for subsequent analysis (Figure 5A). Bray‐Curtis distance‐based principal coordinate analysis illustrated a distinct segregation between the C and HS groups, with the BHBA‐treated group aligning closer to the C group in both horizontal and vertical coordinates (Figure 5B; PERMANOVA: *F* = 3.075, *p* = 0.001). Alpha diversity analysis, employing various indices such as the Chao1 and Shannon indices, revealed a declining trend in the Chao1 index and a significant rise in the Shannon index within the HS group compared to the C group (Figure 5C; *p* > 0.05 and *p* < 0.05, respectively). Interestingly, the alpha diversity indices of the BHBA group were closer to that of group C than the HS group (Figure 5C).

A total of 761 (12.5%) ASVs (Amplicon Sequence Variants) were found in the three groups, and 1605 (26.4%), 1336 (22.0%), and 1440 (23.7%) ASVs were found in the C, HS, and BHBA groups, respectively (Figure 5D). The composition of the gut microbiota community in colonic contents significantly varied among the treatment groups at the phylum (top 10), as depicted in Figure 5E. In the C and BHBA groups, Firmicutes, Bacteroidetes, and Tenericutes dominated at the phylum level, whereas in the HS group, Firmicutes, Bacteroidetes, and Actinobacteria were predominant (Figure 5E). HS treatment increased the abundance of Bacteroidetes, Actinobacteria, Proteobacteria, and TM7 (*p >* 0.05, *p* < 0.01, *p >* 0.05, and *p* < 0.01, respectively) while decreasing the abundance of Firmicutes, Verrucomicrobia (all *p <* 0.05), and the Firmicutes/Bacteroidetes (F/B) ratio (*p >* 0.05) compared to the C group (Figure 5F–H). The BHBA group had the higher relative abundance of Firmicutes (*p >* 0.05), Verrucomicrobia (*p >* 0.05), and F/B ratio (*p >* 0.05) compared to the HS group; whereas the relative abundance of Bacteroidetes, Actinobacteria, Proteobacteria, and TM7 had lower relative abundance (Figure 5F–H; *p* > 0.05, *p <* 0.05, *p <* 0.05, and *p <* 0.01, respectively).

**FIGURE 6:**
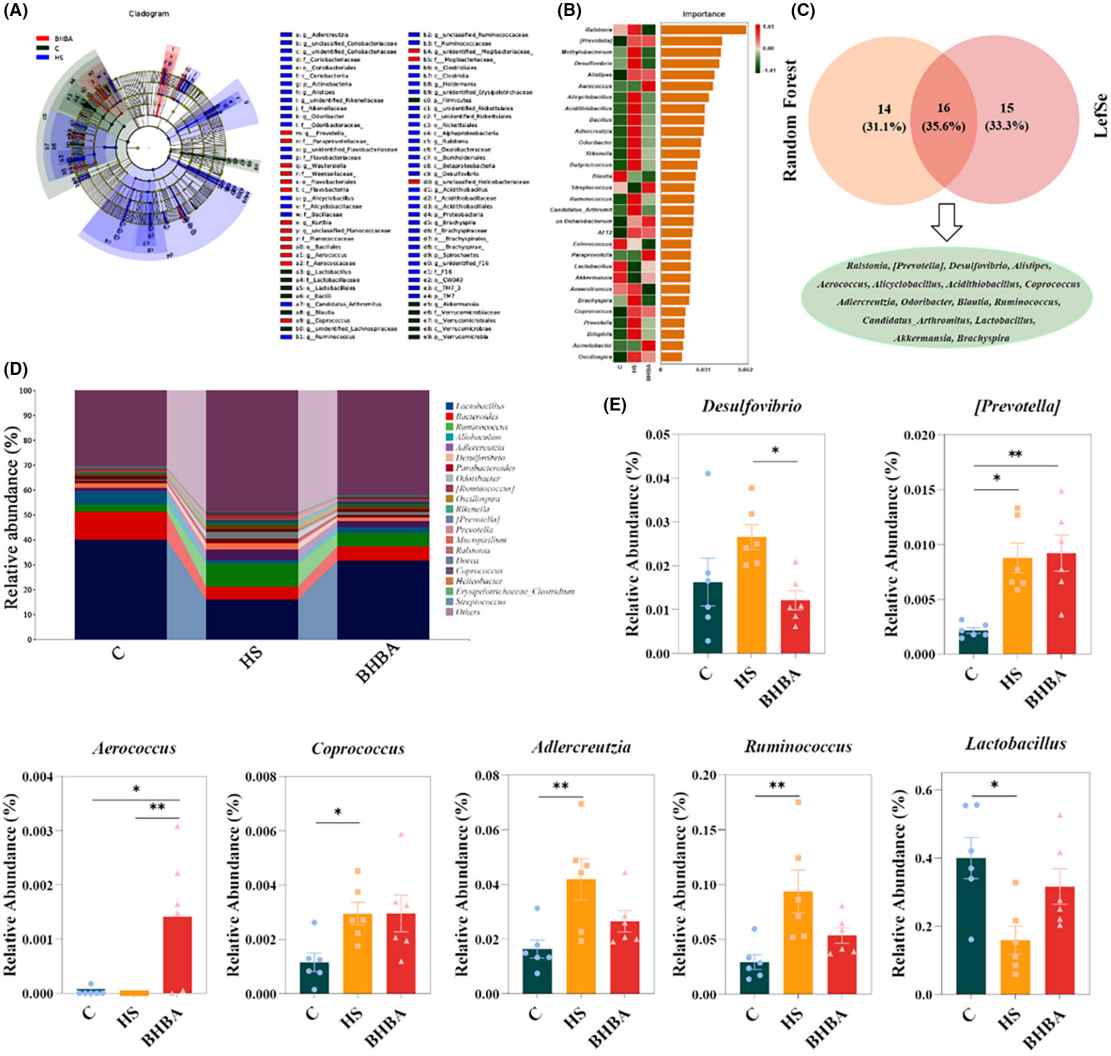
BHBA protects HS‐induced abnormal changes in the gut microbiota at the genus level. (A) Cladogram of LDA effect size (LEfSe). (B) Random forest analysis (top 30 in importance). (C) Venn diagram for the LEfSe and the random forest. (D) Relative abundance of gut microbial composition at the genus level (top 20). (E) The effects of HS and BHBA on the relative abundance of gut microbiota at the genus level (*Desulfovibrio* spp., *[Prevotella]* spp., *Aerococcus* spp., *Coprococcus* spp., *Adlercreutzia* spp., *Ruminococcus* spp., and *Lactobacillus* spp., respectively). *p < 0.05 and **p < 0.01. All data are expressed as the mean ± SEM, *n* = 6. BHBA, β‐Hydroxybutyric acid; HS, heat stress; LDA.

A cladogram was utilized to determine which species were substantially enriched in each group after a genus‐level study of the gut microbiota using LEfSe analysis and a current LDA (Linear Discriminant Analysis) threshold of 2.5. A total of 31 genera were screened (Figure 6A). The microbiota from the most relevant genera (top 30) were then filtered using random forest analysis, and the most important species were *Ralstonia* spp., *[Prevotella]* spp., and *Methylobacterium* spp. (Figure 6B). Moreover, a Venn diagram illustrates genera with LDA values exceeding 2.5 and the top 30 most important genera, identifying 16 species, including *Ralstonia* spp., *[Prevotella]* spp., and *Desulfovibrio* spp. (Figure 6C). Additionally, HS led to a decrease in the relative abundance of *Lactobacillus* spp. (*p <* 0.05) but an increase in *Desulfovibrio* spp. (*p >* 0.05), *[Prevotella]* spp. (*p <* 0.05), *Coprococcus* spp. (*p <* 0.05), *Adlercreutzia* spp. (*p <* 0.01), and *Ruminococcus* spp. (*p <* 0.01) compared to the C group (Figure 6D,E). It was observed that the relative abundance of *Aerococcus* spp. (*p <* 0.05) increased in the BHBA group, while *Coprococcus* spp. (*p >* 0.05) and *Lactobacillus* spp. (*p >* 0.05) showed no significant change. Conversely, *Desulfovibrio* spp. (*p <* 0.05), *Adlercreutzia* spp. (*p >* 0.05), and *Ruminococcus* spp. (*p >* 0.05) exhibited lower abundance in the BHBA group (Figure 6D,E). In comparison to the C group, we found that HS reduced the abundance of bacteria linked with tryptophan (Trp) metabolism, such as *Lactobacillus* spp.^43^

We evaluated the potential of differential taxa at the phylum and genus levels as biomarkers for assessing the occurrence of HS and found that a single differentiating bacterium served as a predictive factor, generating area under the curve (AUC) values ranging from 0.75 to 1.00 and 0.583 to 1.00, respectively (Figure S1a,b in Appendix S1). TM7 and *[Prevotella]* spp. emerged as the optimal discriminant predictor at the phylum and genus levels, respectively (both AUC: 1.00), with the best cutoff values of 0.013 and 0.004, respectively (Figure S1c–f in Appendix S1). Additionally, multivariate stepwise logistic regression analysis demonstrated that the combination of indicators at both the phylum and genus levels exhibited superior prediction performance (both AUC: 1.00) (Figure S1g,h in Appendix S1). These findings suggest that TM7 and *[Prevotella]* spp. may serve as the best potential biomarkers for evaluating the occurrence of HS, whereas BHBA significantly reversed HS‐induced gut microbiota dysbiosis in mice.

### 3.5 BHBA alleviates neuroinflammation via the gut–brain axis

**FIGURE 7:**
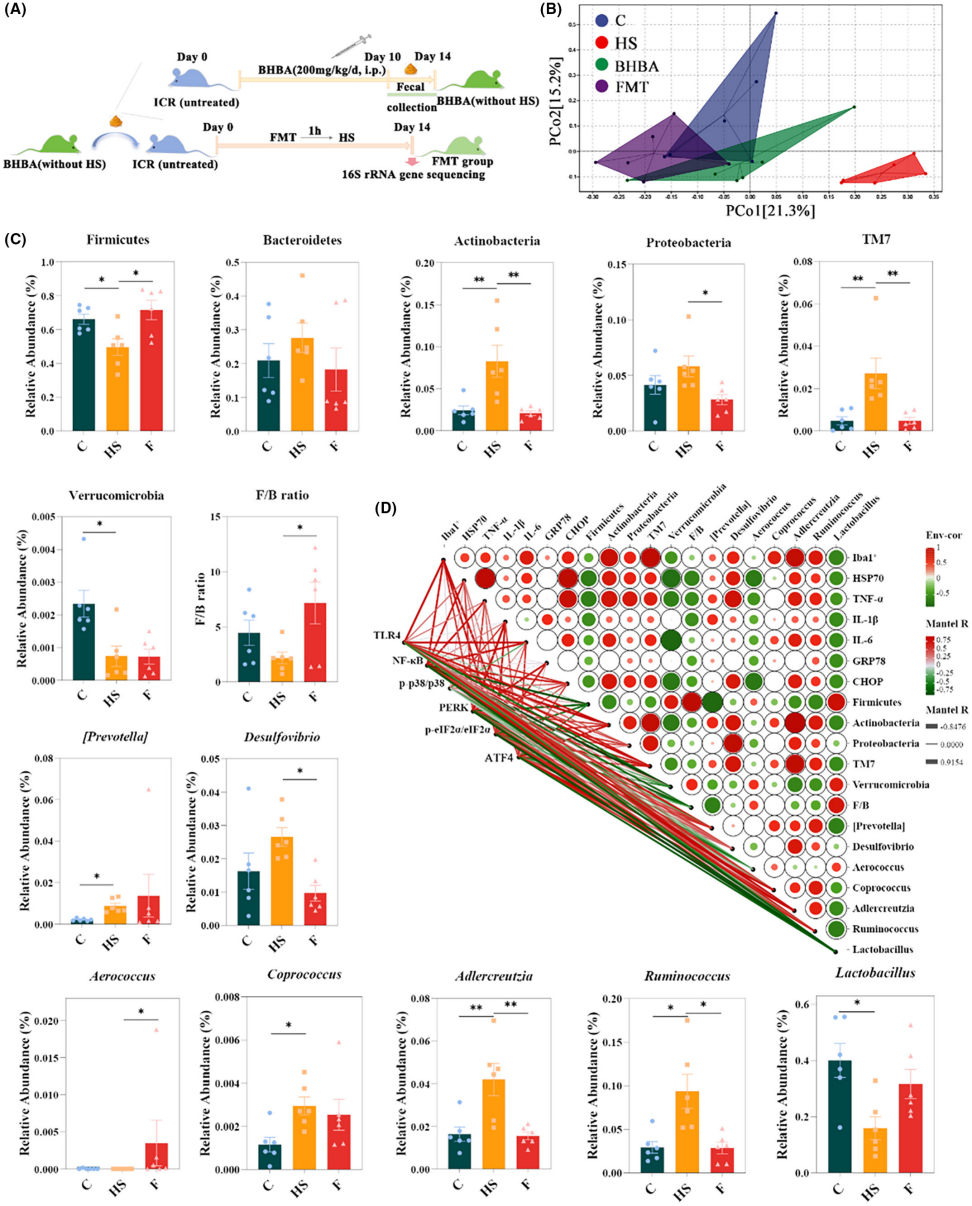
FMT modeling and the effect of gut microbiota on neuroinflammation and ERS. (A) Establishment of the FMT model. (B) Bray‐Curtis distance‐based PCoA estimates for the gut microbiota of the C, HS, BHBA, and FMT groups. (C) The effects of HS and FMT on the relative abundance of gut microbiota (Firmicutes, Bacteroidetes, Actinobacteria, Proteobacteria, TM7, Verrucomicrobia, F/B ratio, *[Prevotella]* spp., *Desulfovibrio* spp., *Aerococcus* spp., *Coprococcus* spp., *Adlercreutzia* spp., *Ruminococcus* spp., and *Lactobacillus* spp., respectively). (D) The heatmap on the right shows the results of Spearman's analysis of the correlations between the indicators of HS, neuroinflammation, ERS, and gut microbiota. The heatmap on the left shows the results of Spearman's analysis of the correlations of TLR4, NF‐κB, p‐p38/p38, PERK, p‐eIF2α/eIF2α, and ATF4. *p < 0.05 and **p < 0.01. All data are expressed as the mean ± SEM, *n* = 6. BHBA, β‐Hydroxybutyric acid; ERS, endoplasmic reticulum stress; FMT, fecal microbiota transplantation; HS, heat stress; PCoA.

FMT has emerged as a promising therapeutic approach for various diseases such as *Clostridium difficile* infection, metabolic syndrome, and neurological disorders by targeting ecological dysbiosis of the gut microbiota.^44^, ^45^, ^46^ Particularly in the therapeutic aspect of neurological disorders, FMT can alleviate the progression or symptoms of neurological disorders by modulating pathways associated with the gut microbiota. For example, Sun et al. showed that FMT administration attenuated an MPTP‐induced mouse model of PD (Parkinson's Disease) by reducing gut microbiota ecological dysbiosis and neuroinflammation.^47^ To investigate whether BHBA alleviation of HS‐induced neuroinflammation in mice is relevant to changes in the gut microbiota, we evaluated the influence of FMT on the gut microbiota of mice by FMT experiments (Figure 7A). The β diversity results are presented using Bray–Curtis distance in the principal coordinate analysis plot, which revealed a clear separation between the HS and FMT groups and overlaps between the FMT, C, and BHBA groups (Figure 7B; PERMANOVA: F = 2.387, *p* = 0.001). Additionally, assessments at both the phylum and genus levels revealed alterations in the gut microbiota compositions of the HS group following FMT (Figure S2a,b in Appendix S1). At the phylum level (Firmicutes, Bacteroidetes, Actinobacteria, Proteobacteria, TM7, and Verrucomicrobia) and genus level (*[Prevotella]* spp., *Desulfovibrio* spp., *Aerococcus* spp., *Coprococcus* spp., *Adlercreutzia* spp., *Ruminococcus* spp., and *Lactobacillus* spp.), the relative abundance of the FMT group in comparison to the HS group displayed similar trends to the BHBA group (Figures 5G,H, 6E, and 7C). The FMT group had a significantly higher F/B ratio (*p* < 0.05) than the HS group, which was identical to the BHBA group (Figures 5F and 7C). These findings indicate that FMT effectively reversed the disruption of gut microbiota in HS mice.

Furthermore, Spearman's correlation analysis was performed to investigate the correlation between HS, neuroinflammation, ERS, and gut microbiota, and the correlation between the TLR4/NF‐κB; TLR4/p38 MAPK; PERK/eIF2α/ATF4/CHOP signaling pathways and gut microbiota (Figure 7D). The heatmap revealed a significant correlation between HSP70, TNF‐α, IL‐6, Iba1+, and CHOP levels and Actinobacteria abundance (*r* = 0.729, 0.800, 0.683, 0.867, 0.763; *p =* 0.026, 0.010, 0.042, 0.002, and 0.017, respectively). TNF‐α mRNA expression level was positively correlated with the abundances of Proteobacteria, TM7, *Desulfovibrio* spp., and *Adlercreutzia* spp. (*r* = 0.750, 0.683, 0.833, and 0.700; *p =* 0.020, 0.042, 0.005, and 0.036, respectively). The density of Iba1+ cells was positively correlated with TM7, *Coprococcus* spp., *Adlercreutzia* spp., and *Ruminococcus* spp. (*r* = 0.933, 0.667, 0.900, and 0.717; *p* = 0.0002, 0.050, 0.001, and 0.030, respectively). The relative abundance of *Desulfovibrio* spp. exhibited positive correlations with the levels of HSP70 and CHOP (*r* = 0.712 and 0.729; *p* = 0.031 and 0.026, respectively). The relative abundance of *Adlercreutzia* spp. was also positively correlated with levels of IL‐6 and CHOP (*r* = 0.667 and 0.695; *p* = 0.050 and 0.038, respectively). Conversely, the relative abundance of Verrucomicrobia displayed negative correlations with the protein expression levels of HSP70 (*r* = −0.780, *p* = 0.013), IL‐6 (*r* = −0.867, *p* = 0.002), and CHOP (*r* = −0.729, *p* = 0.026). Similarly, the relative abundance of *Aerococcus* spp. exhibited negative correlations with the protein expression levels of HSP70 (*r* = −0.687, *p* = 0.041) and CHOP (*r* = −0.724, *p* = 0.027).

Furthermore, TLR4 protein expression levels exhibited positive correlations with the relative abundance of Actinobacteria (*r* = 0.712, *p* = 0.031) and *Adlercreutzia* spp. (*r* = 0.763, *p* = 0.017). NF‐κB exhibited positive correlations with the relative abundance of Actinobacteria, TM7, *[Prevotella]* spp., *Adlercreutzia* spp., and *Ruminococcus* spp. (*r* = 0.915, 0.763, 0.729, 0.881, and 0.729; *p* = 0.001, 0.017, 0.026, 0.002, and 0.026, respectively), while showing negative correlations with Firmicutes, Verrucomicrobia, and *Lactobacillus* spp. (*r* = −0.780, −0.848, and −0.729; *p* = 0.013, 0.004, and 0.026, respectively). PERK was negatively correlated with the relative abundance of *Lactobacillus* spp. (*r* = −0.780, *p* = 0.013, respectively) and F/B (*r* = −0.695, *P* = 0.038, respectively). The level of ATF4 protein expression was positively correlated with Actinobacteria (*r* = 0.780, *p* = 0.013), TM7 (*r* = 0.678, *p* = 0.045), *Coprococcus* spp. (*r* = 0.797, *p* = 0.010), and *Adlercreutzia* spp. (*r* = 0.831, *p* = 0.006), while negatively correlated with the relative abundance of *Lactobacillus* spp. (*r* = −0.678, *p* = 0.045). Based on these results, the inhibition of neuroinflammation under heat stress by BHBA probably functions through the gut‐brain axis.

## 4 DISCUSSION

Heat stress is a widespread global environmental problem and an important environmental factor in the development of psychiatric diseases.^48^ Using mouse and BV2 cell models, we demonstrated that the development of HS may be associated with ERS and neuroinflammation. The mechanism of BHBA's action on HS‐induced gut microbial dysbiosis and neuroinflammation has not been well elucidated, although previous pharmacological experiments have demonstrated neuroprotective and anti‐inflammatory activities of BHBA.^22^, ^49^ We observed that BHBA could inhibit HS by remodeling the gut microbiota and attenuating ERS‐dependent neuroinflammation.

Inflammation is triggered by immunogenic stimuli (i.e., pathogens or tissue injury), leading to a variety of biochemical cascades designed to resolve these threats to homeostasis in the body; however, aberrant inflammatory processes in the central nervous system can result in neurological impairment, for example, chronic inflammation impairs neuronal function and leads to cell death.^50^, ^51^, ^52^ According to our experimental results, the cerebral cortex of HS mice exhibited a pronounced inflammatory response, which was successfully inhibited by BHBA. Meanwhile, in BV2 cells, BHBA concurrently demonstrated a strong suppression of the inflammatory response triggered by HS.

Microglia are increasingly recognized as pivotal regulators of neuronal function and behavior across various domains of neuroscience, making them a focal point of research in neurological disorders, particularly neuroinflammation.^53^, ^54^ As macrophages in the brain, microglia are uniquely positioned to regulate neuro homeostasis and behavior. Recent studies have demonstrated that microglia play a role in regulating neuronal activity, facilitating learning, and influencing social behavior.^55^, ^56^ There is growing evidence that the pathogenesis of HS‐associated neuroinflammation is closely linked to microglia in the brain. Huang et al. found that HS can induce excessive activation of microglia and astrocytes during the generation of neuroinflammation.^31^ In an early study of HS on the inflammatory response in the anterior hypothalamus (AH) of chicks, it was found that HS altered the reactivity of hypothalamic microglia as manifested by suppression of microglia in response to LPS stimulation.^57^ Another important issue during the assessment of microglia status, in addition to morphological approaches, is the reliance on Iba‐1 immunoreactivity to report their activation status.^58^ Interestingly, our study found a considerable increase in microglial cell overactivation and proliferation in the HS animal model, which was alleviated by BHBA therapy. This is consistent with BHBA's inhibitory action on HS‐induced neuroinflammation in BV2 cells.

It is well known that ER is a major subcellular organelle for synthesizing and folding proteins, and the production of ROS is a factor that endangers ER in the area of protein folding, leading to the accumulation of unfolded or misfolded proteins.^59^ Our study uncovered a notable rise in microglial cell overactivation and proliferation in the HS animal model, both of which were alleviated by BHBA treatment.^60^, ^61^ Numerous studies have reported that neuroinflammation is closely related to ERS. For example, Zhang et al. discovered that inhibiting MALT1 reduced spinal cord ischemia/reperfusion injury‐induced neuroinflammation in rats by regulating glial ERS.^62^ Zeng et al. discovered that the autophagy protein NRBF2 increases autophagosome formation by interacting with Rab7 during subarachnoid hemorrhage, reducing ERS‐related neuroinflammation and oxidative stress.^63^ ERS can activate UPR through three pathways to maintain protein homeostasis, including IRE1/XBP1, PERK‐eIF2α‐ATF4, and ATF6.^64^ In general, GRP78 interacts with transmembrane proteins (PERK, IRE1, and ATF6) to keep them stable and non‐reactive. When damage causes ERS, GRP78 separates from them and initiates the UPR stress response.^65^ Under normal conditions, CHOP maintains low cytoplasmic expression levels; however, during ERS, GRP78 dissociates from endoplasmic reticulum transmembrane proteins PERK, ATF6, and IRE1, thereby triggering CHOP activation.^66^ Therefore, GRP78 and CHOP are considered important markers of ERS.^67^ Consistent with prior findings, we observed heightened GRP78/CHOP expression in both HS mice and BV2 cells, indicating the presence of ERS in both in vivo and in vitro HS models, with BHBA exhibiting inhibitory effects on these processes.^31^

Among the three pathways involved in the ERS process, activation of the PERK pathway is initiated by the phosphorylation of PERK, followed by the formation of the corresponding dimer, which then phosphorylates downstream elF2α. p‐elF2α can promote the translation of the transcription factor ATF4, and when the ERS is too severe, ATF4 initiates the transcription of CHOP, which initiates a pro‐apoptotic program that promotes apoptotic cell death.^68^ To elucidate the mechanism underlying ERS in HS, we assessed the expression levels of PERK, eIF2α, p‐eIF2α, ATF4, and CHOP. Remarkably, expression levels of PERK, p‐eIF2α/eIF2α, ATF4, and CHOP were higher in the HS group compared to the C group. This phenomenon was mitigated by BHBA treatment. These findings imply that ERS occurs in both mouse and cellular HS models, and BHBA may mitigate ERS through the PERK/eIF2α/ATF4/CHOP pathway.

During the ERS response, both PERK and IRE1/XBP1 trigger inflammatory signaling.^69^, ^70^ Among them, in addition to attenuating translational functions, the sustained activation of PERK also leads to deterioration of the mitochondrial stress response and activation of the NF‐κB/TNFα axis, which ultimately determines the fate of the cell.^71^, ^72^, ^73^ It has been reported that eIF2α, a downstream signal of PERK, can activate NF‐κB by decreasing the level of IκBα in mouse embryonic fibroblasts^74^; in recent years, researchers have discovered a pathway independent of classical IκBα, in which PERK kinase transmits stress signals from the endoplasmic reticulum to the nucleus via the PERK/STAT3/NF‐κB/TNFα axis under long‐term E2‐deprived (LTED) conditions.^73^ Besides, as an upstream protein activated by inflammatory responses, TLR4 is widely expressed in microglia, and NF‐κB is an important factor downstream of the TLR4 signaling pathway, which is involved in the regulation of immune responses.^75^, ^76^ Therefore, when TLR4 activates NF‐κB, it can also produce a large number of inflammatory factors, inducing an inflammatory cascade response that results in neuroinflammation.^77^, ^78^

Interestingly, activated PERK also activates p38MAPKase, in which p38 dissociates from Hsp90, which inhibits PERK, and undergoes autophosphorylation. Activated p38 then further enhances the docking of activating transcription factor 4 (ATF4) to the CHOP promoter via eIF2α, thereby enhancing apoptosis.^75^ To explore the correlation between neuroinflammation and ERS in HS, we also assessed the expression of TLR4, NF‐κB, and p‐p38/p38. Notably, both HS mice and BV2 cells exhibited elevated levels of TLR4, NF‐κB, and p‐p38/p38. These findings indicate the occurrence of ERS in both animal and cellular HS models, potentially triggering the activation of NF‐κB and p38 inflammatory pathways via PERK, thereby leading to neuroinflammation. Moreover, BHBA demonstrated an inhibitory effect on ERS activation and inflammatory signaling pathways in both animal and cellular HS models.

The human colon harbors the largest portion of the intestinal commensal microbiota, constituting a dynamic and intricate system that necessitates sustained barriers and modulatory mechanisms to uphold host–microbe interactions, tissue integrity, immunological equilibrium, and overall individual physiology.^79^ Among the myriad factors influencing the gut microbiota, HS significantly impacts the composition and functionality of microorganisms.^41^, ^42^ Numerous studies have indicated that decreased F/B ratios correlate with individual weight loss, aligning with earlier observations of weight reduction in mice under HS conditions.^31^, ^80^, ^81^ BHBA serves as both a metabolic intermediary for physiological fuels and a signaling molecule that regulates energy expenditure and homeostasis during nutritional deficiency.^82^ For instance, BHBA inhibited lipolysis in mouse and human adipose tissue; dietary supplementation with both low and high doses of BHBA increased body weight in early weaned goats and had a positive effect on BHBA on growth performance, organ development, and health in response to weaning stress in early weaned goats.^83^, ^84^, ^85^ Therefore, the F/B ratios reflect to some extent the regulatory influences of HS and BHBA on body weight in mice.

At the phylum level, we observe a significantly higher relative abundance of TM7, which is identified as the most discriminative predictor compared to the C group. To date, TM7 is a commensal member of the oral, gastric, skin, and intestinal microbial communities of healthy individuals.^86^, ^87^, ^88^, ^89^ According to studies utilizing 16S rRNA analysis, TM7 thrives in inflammatory environments, exhibiting increased relative abundance in conditions, such as vaginosis, various lung diseases, and inflammatory bowel disease.^86^ Several rodent model studies have further associated gastrointestinal diseases with TM7, with increased representation of TM7 in colitis, irritable bowel syndrome, and colon cancer.^86^ TM7 has been identified as a potentially causative bacterial group capable of initiating or exacerbating the progression of periodontitis.^90^, ^91^ However, current research suggests that in a mouse model of ligature‐induced periodontitis, TM7 may be able to attenuate inflammation and consequent bone loss by altering the pathogenicity of host bacteria.^86^ Studies on the correlation between HS and TM7 are extremely Limited; Liu et al. found that TM7 abundance was lower in the jejunum of heat‐stressed yellow‐feathered broilers and that its abundance was significantly correlated with the expression of immune‐related genes.^92^ However, further study is needed to determine the relationship between TM7 and HS. We analyzed the gut microbiota at the genus level, using a current LDA threshold of 2.5, and identified the top 30 species in terms of importance. In this study, the microbiota of mice stimulated by high temperatures was found to differ significantly between the HS group and the C group in three genera: *Lactobacillus* spp., *Ruminococcus* spp., and *[Previotella]* spp. Notably, *Lactobacillus* spp. is significantly correlated with tryptophan. Previous research has demonstrated that specific *Lactobacillus* strains in the host facilitate memory behavior by metabolizing tryptophan into indole derivatives, which activate the host aryl hydrocarbon receptor.^93^ Moreover, *Lactobacillus* spp. has been associated with HS, and Deng et al. found that *Lactobacillus* spp. could treat HS‐induced lipid metabolism disorders.^94^ *Ruminococcus* spp. is closely associated with inflammatory bowel disease and neurological disorders. Among them, Ruminococcus gnavus is a common member of the human gut microbiota, which is abundant in inflammatory bowel disease and neurological diseases.^95^, ^96^, ^97^ Research has shown that the metabolites of Ruminococcus gnavus can affect brain regulation and function by influencing adult hippocampal granule cell development and synaptic plasticity.^98^ Most *Prevotella* spp. are associated with humans and have been found inhabiting multiple body parts, including the skin, mouth, vagina, and gastrointestinal tract.^99^ They are key players in the balance between health and disease. Although *Prevotella* spp. do not contain specific pathogenic species known to cause disease; their members have been associated with a variety of diseases, including inflammatory autoimmune diseases,^100^ opportunistic infections,^101^ bacterial vaginosis,^102^ and oral biofilm formation and disease.^103^, ^104^ However, more evidence is needed regarding the relationship between *Prevotella* spp. and neurologic‐related symptoms during HS.

Through FMT and Spearman's correlation analysis, our results suggest that the improvement of BHBA on HS may play a role in alleviating ERS‐induced neuroinflammation by regulating the gut microbiota of the colon through the gut‐brain axis. Wang et al. also found that dimethylglycine could alleviate HS‐induced intestinal barrier damage by regulating the cecum microbial community and improving the metabolic function of the gut–brain axis (intestinal tryptophan metabolism and hypothalamic tryptophan metabolism) in their study of HS.^105^ Therefore, regulatory strategies through the gut–brain axis may be an effective way to mitigate the negative effects of HS on human and animal health. At present, the neuroprotective effects of BHBA have been shown to be mediated by GPR109A, which regulates monocyte/macrophage function and redirects these cells to neuroprotective pathways.^106^ Moreover, GPR109A is a regulator of bacterial flora action, which is importantly characterized by colony‐dependence in the colon and ileum.^107^ In recent years, GPR109A has also been implicated in the regulation of intestinal metabolites, suppression of colonic inflammation, and maintenance of intestinal barrier integrity.^108^, ^109^ Previous studies have demonstrated that microbiota‐derived butyrate and NA, acting as ligands for GPR109A receptors, suppress inflammation by activating GPR109A in intestinal epithelial cells, dendritic cells, and macrophages.^110^ Hence, investigating whether GPR109A is involved in regulating the host gut microbiota during HS and whether it can mitigate neuroinflammation by modulating the gut–brain axis during BHBA treatment may be an important direction for future research.

Behavioral changes in animals are important indicators for assessing neurological function. The main limitation of the present study is the lack of assessment of the behavioral effects in animals, thus preventing a more comprehensive understanding of the overall effects of BHBA in modulating heat stress‐induced neuroinflammation, including its potential impact at the behavioral level. However, the present study validated the neuroprotective effects of BHBA from multiple perspectives through in vivo experiments combined with in vitro cellular modeling, providing a theoretical basis for BHBA as a prophylactic drug with potential clinical applications for the treatment of neurological disorders. Meanwhile, exploring whether GPR109A is involved in the regulation of the host gut microbiota during HS and whether it can attenuate neuroinflammation by regulating the gut‐brain axis during BHBA treatment may be an important direction for future research.

## 5 CONCLUSION

In conclusion, BHBA affects the development of HS‐induced neuroinflammation through the regulation of the gut‐brain axis. Specifically, BHBA regulates gut microbiota homeostasis mainly by acting on TM7, *Lactobacillus* spp., *Ruminococcus* spp., and *[Prevotella]* spp. in the colon. BHBA regulates HS‐induced ERS by acting on PERK/eIF2α/ATF4/CHOP and modulates neuroinflammation through the TLR4/NF‐κB and TLR4/p38MAPK signaling pathways. Our findings suggest that the gut‐brain axis mediated by gut microbiota is a novel idea for addressing HS‐related neurological disorders.

## AUTHOR CONTRIBUTIONS

XF, YM, and JH performed the experiments. XYZ and JGW conceived and designed the study. YZS wrote the manuscript. YMZ and YLL contributed to the statistical analysis. JGW and XYZ revised the manuscript. All authors read and approved the final manuscript.

## CONFLICT OF INTEREST STATEMENT

The authors have no conflicts of interest to declare.

## Supporting information


Appendix S1

Appendix S2

## Data Availability

Raw Illumina read data of 16S rRNA gene sequencing were uploaded to the NCBI Sequence Read Archive (PRJNA 1071825). The data sets generated during this study are available from the corresponding author upon reasonable request.
