# Supplementary material for: BHBA attenuates endoplasmic reticulum stress‐dependent neuroinflammation via the gut–brain axis in a mouse model of heat stress
Source: CNS Neurosci Ther. 2024 Jul 7;30(7):e14840. doi: 10.1111/cns.14840 (PMC11228358; doi:10.1111/cns.14840)
Supplement: Supplementary file 1 — Appendix S1 [file CNS-30-e14840-s002.docx]

**Supplementary Information**

**Additional file 1:**

| Gene | Gene ID | Sequence（5’-3’） | length (bp) |
| --- | --- | --- | --- |
| Gapdh | [NM_001411843.1](https://www.ncbi.nlm.nih.gov/entrez/viewer.fcgi?db=nucleotide&id=2295558070) | F:AGGTTGTCTCCTGCGACTCCA  R:GTGGTCCAGGGTTTCTTACTCC | 185 |
| TNF-α | [NM_001278601.1](https://www.ncbi.nlm.nih.gov/entrez/viewer.fcgi?db=nucleotide&id=518831588) | F:AGTCCGGGCAGGTCTACTTT  R:GTCACTGTCCCAGCATCTTGT | 231 |
| IL-1β | XM_006498795.5 | F:TGACGGACCCCAAAAGATGA  R:TCTCCACAGCCACAATGAGT | 140 |
| IL-6 | [NM_001314054.1](https://www.ncbi.nlm.nih.gov/entrez/viewer.fcgi?db=nucleotide&id=930945755) | F:CACTTCACAAGTCGGAGGCT  R:CTGCAAGTGCATCATCGTTGT | 113 |
| IL-10 | NM_010548.2 | F:GTAGAAGTGATGCCCCAGGC  R:CACCTTGGTCTTGGAGCTTATT | 187 |

**Table S1. Primer sequences for qPCR analysis.**Gapdh: Glyceraldehyde-3-phosphate dehydrogenase; TNF-α: Tumor necrosis factor-α; IL-1β: Interleukin-1β; IL-6: Interleukin-6; IL-10: Interleukin-10.

**
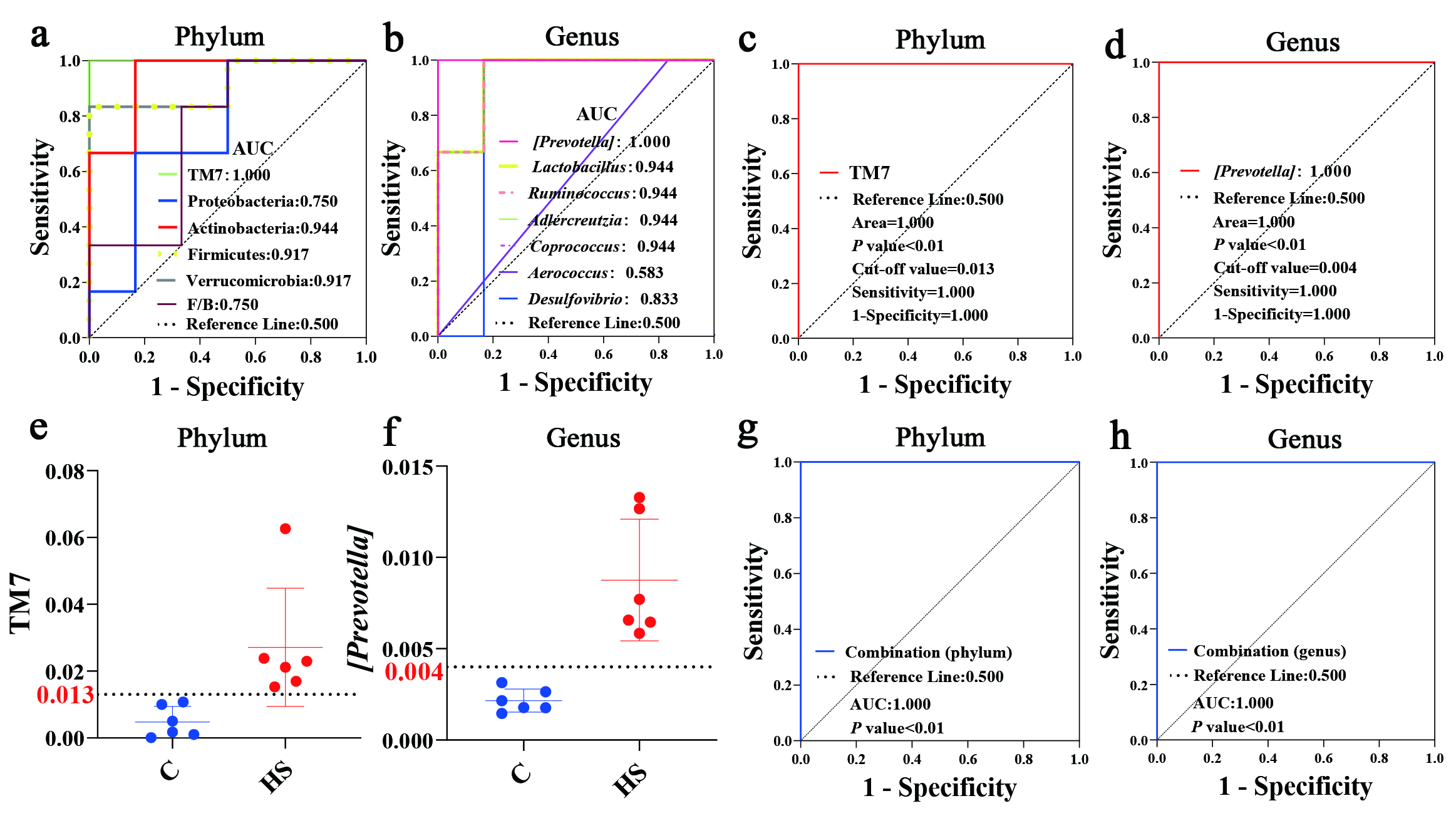
**

**Figure S1. Diagnostic value assessment of the gut microbiota.**

1. ROC curves (including Firmicutes, Actinobacteria, Proteobacteria, TM7, Verrucomicrobia, F/B ratio) at the phylum level (n=6). **(b)** ROC curves (including *[Prevotella]* spp., *Desulfovibrio* spp., *Aerococcus* spp., *Coprococcus* spp., *Adlercreutzia* spp., *Ruminococcus* spp., and *Lactobacillus* spp.) at the genus level (n=6). **(c, d)** ROC curve and best cut-off value of TM7 (n=6). **(e, f)** ROC curve and best cut-off value of *[Prevotella]* spp. (n=6). **(g)** ROC curve of combination at the phylum level (n=6). **(h)** ROC curve of combination at the genus level (n=6).


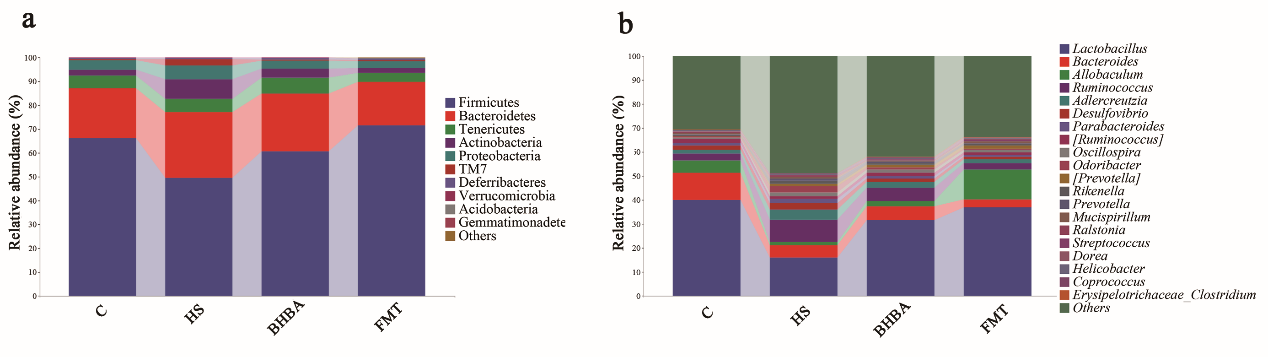
**Figure S2. Relative abundance of gut microbial composition in groups C, HS, BHBA, and FMT.**

**(a)** The phylum level (top 10) (n=6). **(b)** The genus level (top 20) (n=6).
