# Supplementary material for: BHBA attenuates endoplasmic reticulum stress‐dependent neuroinflammation via the gut–brain axis in a mouse model of heat stress
Source: CNS Neurosci Ther. 2024 Jul 7;30(7):e14840. doi: 10.1111/cns.14840 (PMC11228358; doi:10.1111/cns.14840)

Full unedited blot for Figure 2h

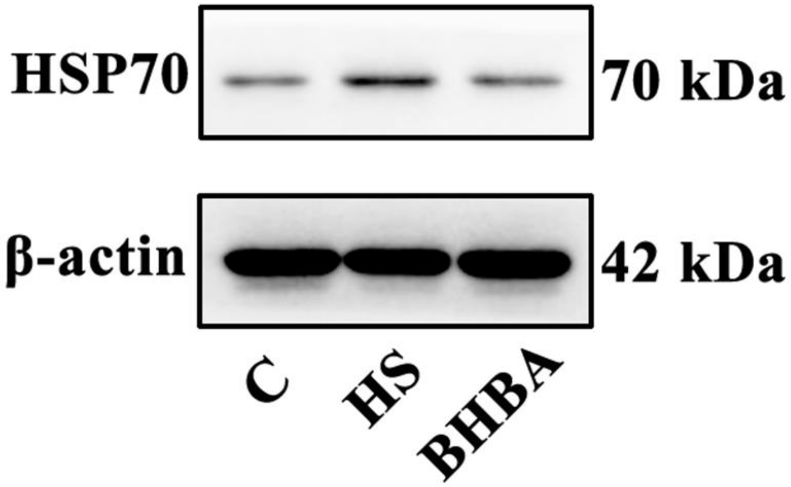

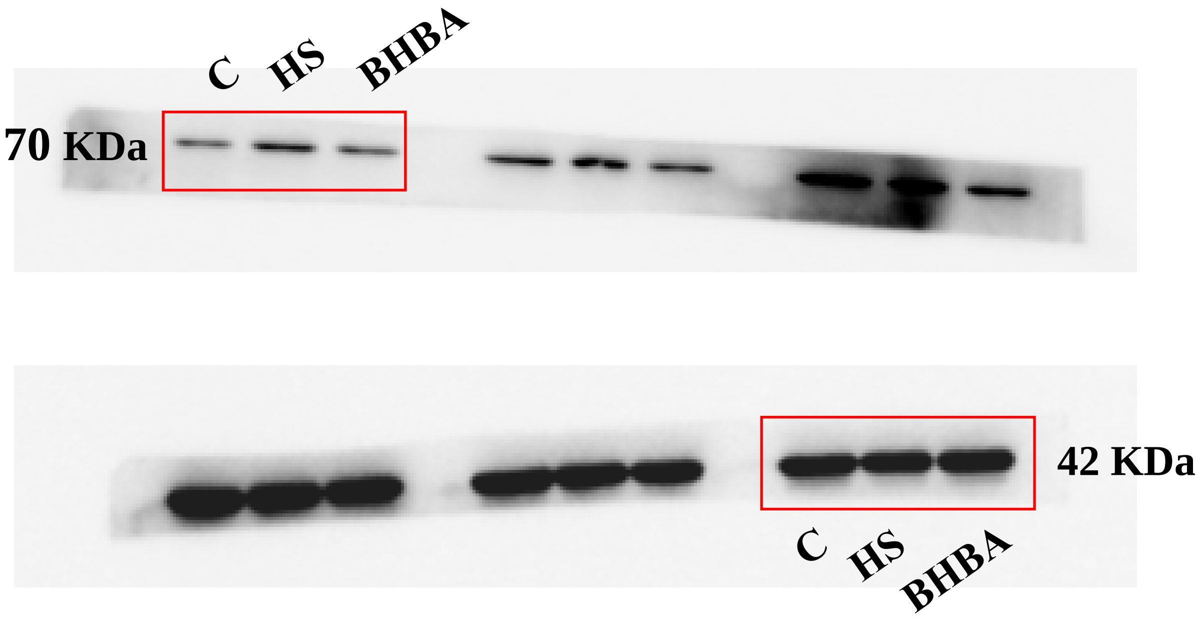

Full unedited blot for Figure 3i (Cerebral cortex)

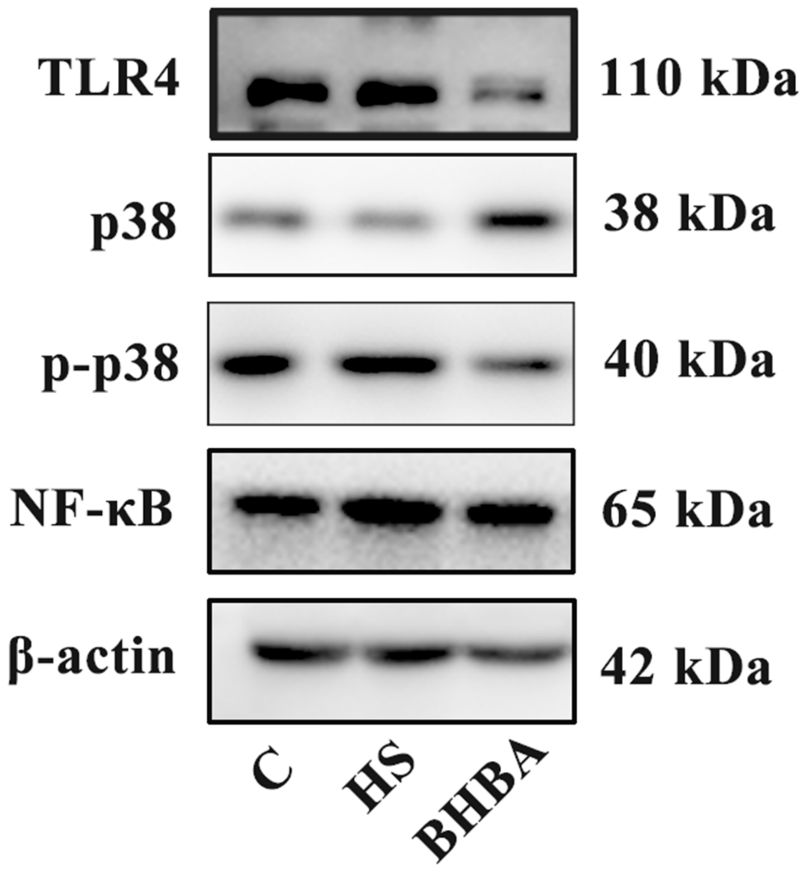

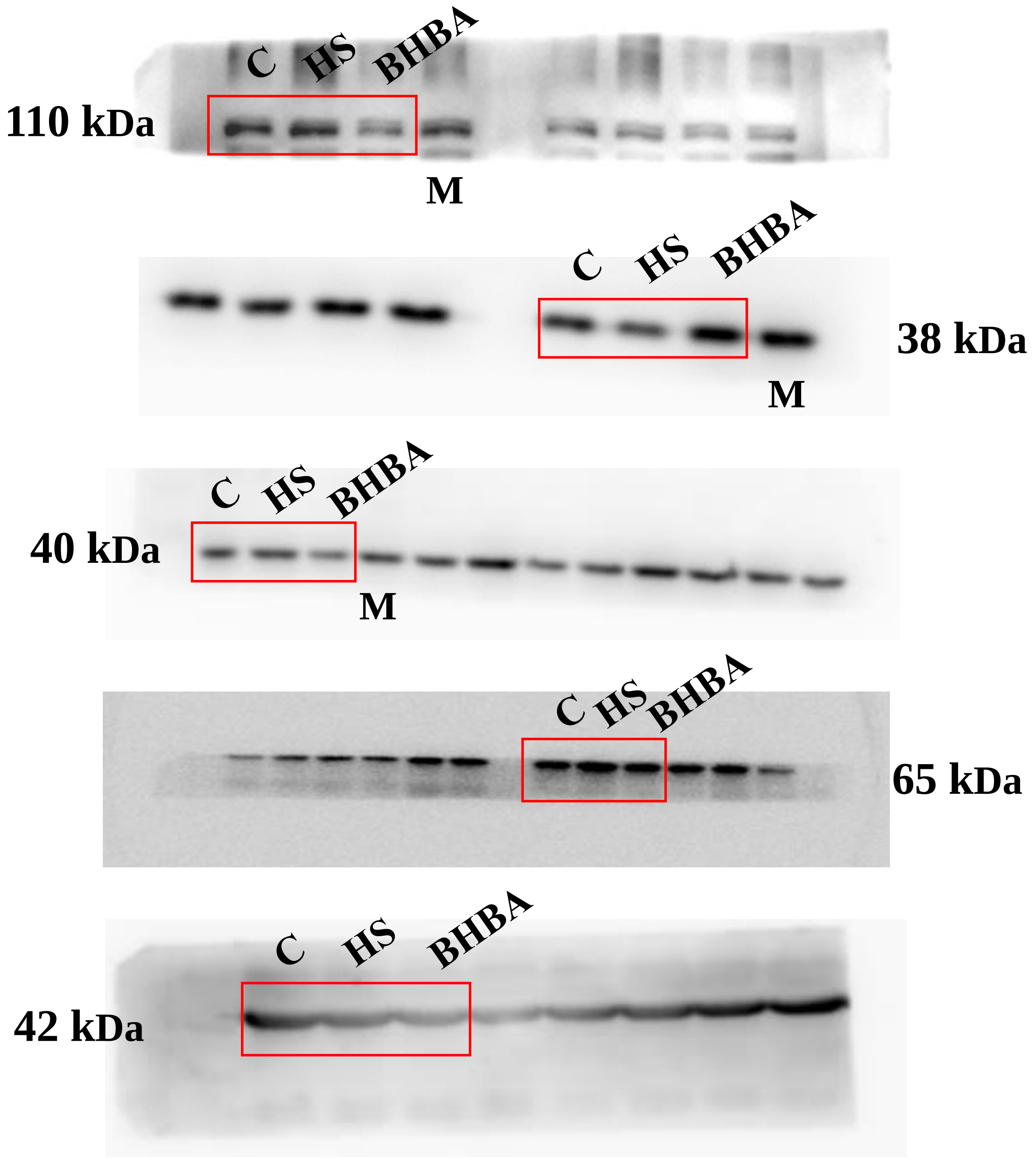

Full unedited blot for Figure 3p (BV2)

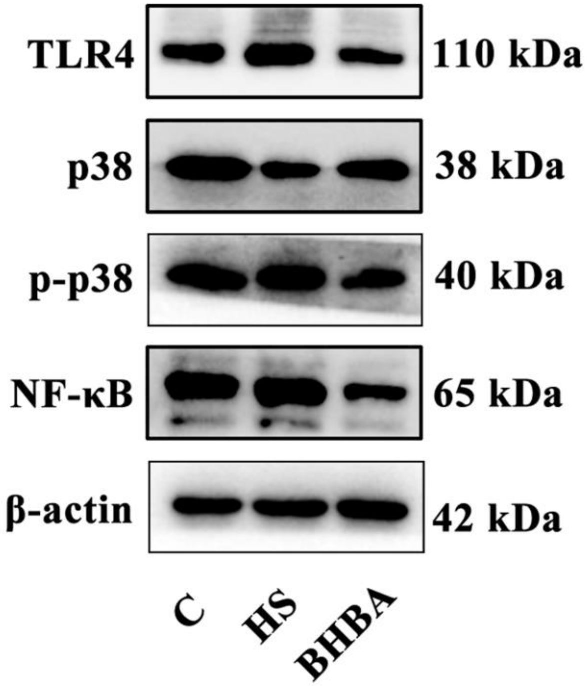

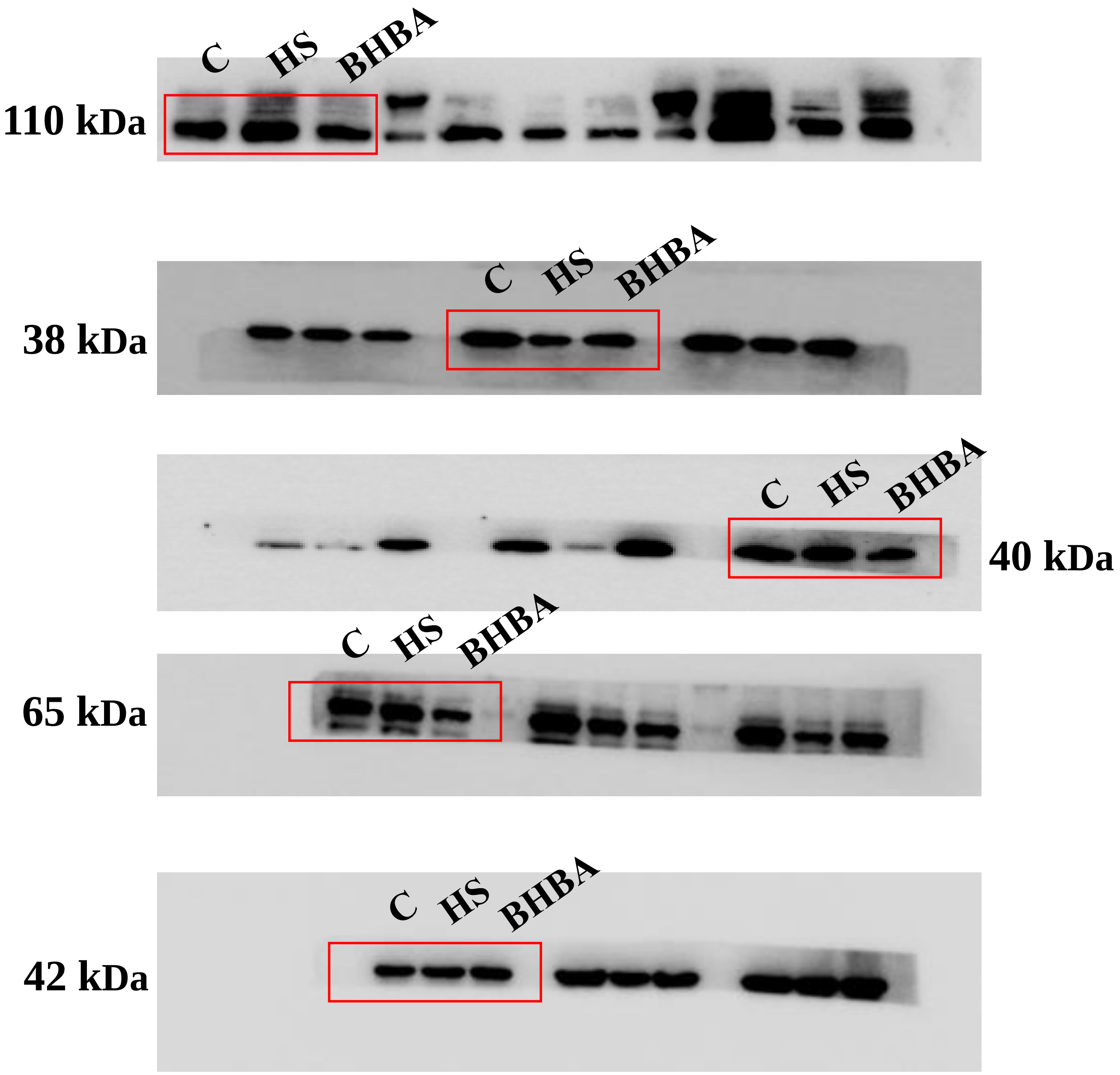

Full unedited blot for Figure 4a (Cerebral cortex)

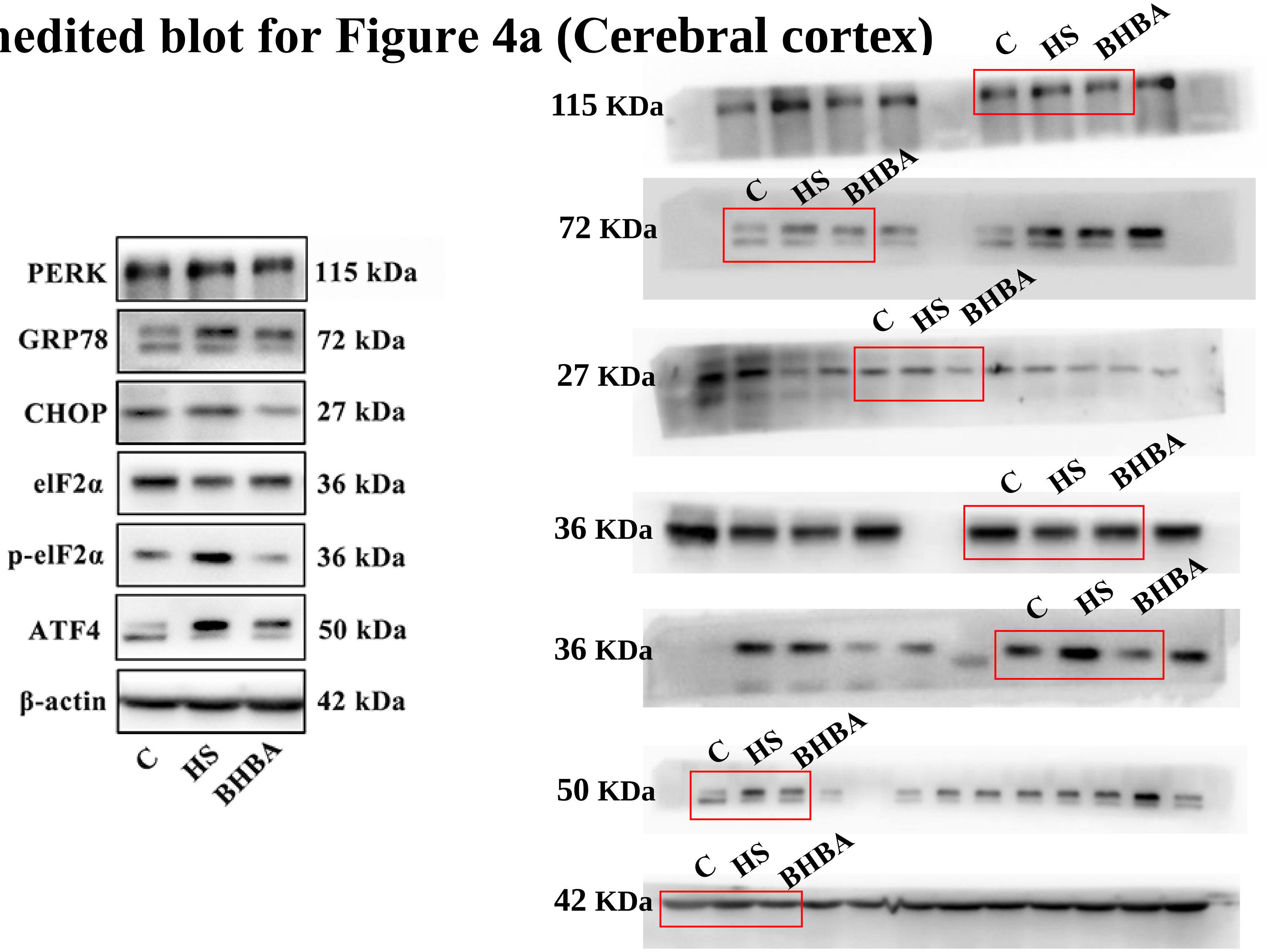

Full unedited blot for Figure 4c (BV2)

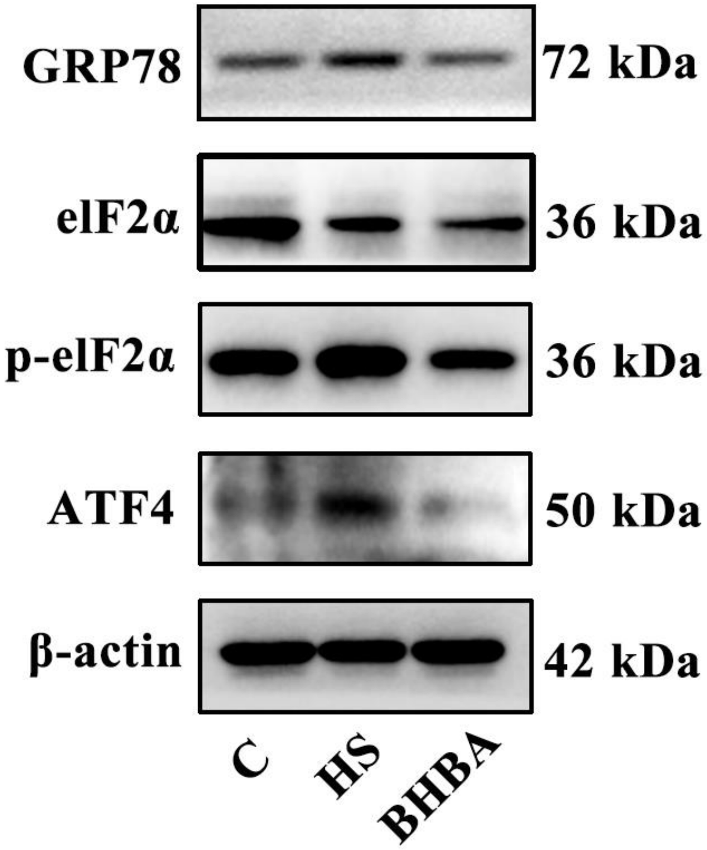

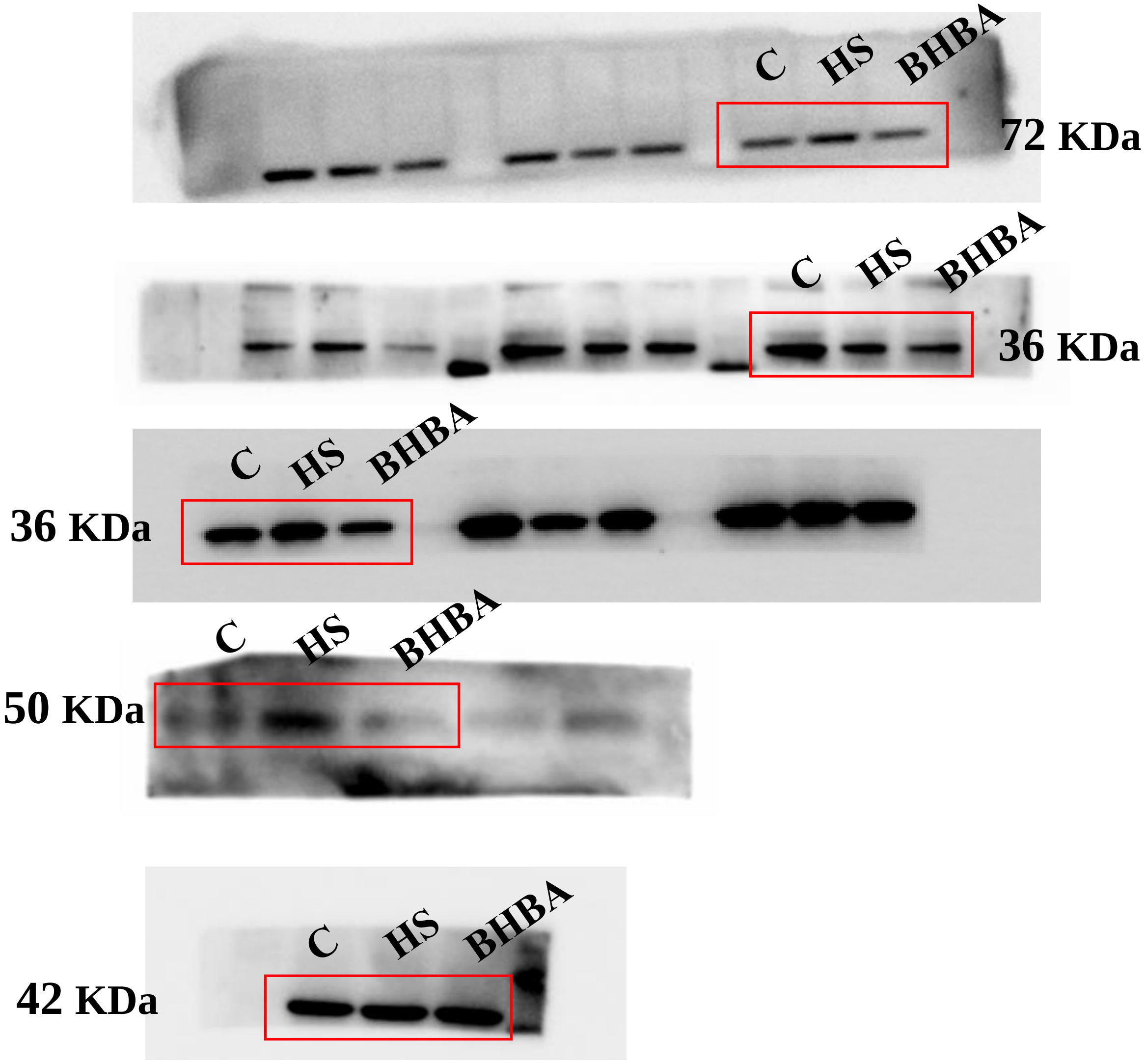

Supplement: Supplementary file 2 — Appendix S2 [file CNS-30-e14840-s001.pdf]
